# Biphasic Mathematical Model of Cell–Drug Interaction That Separates Target-Specific and Off-Target Inhibition and Suggests Potent Targeted Drug Combinations for Multi-Driver Colorectal Cancer Cells

**DOI:** 10.3390/cancers12020436

**Published:** 2020-02-13

**Authors:** Jinyan Shen, Li Li, Tao Yang, Paul S. Cohen, Gongqin Sun

**Affiliations:** 1 Department of Cell and Molecular Biology, University of Rhode Island, Kingston, RI 02881, USA; jinyanshen201@163.com (J.S.); lili_5076@126.com (L.L.); yangtao056cn@126.com (T.Y.); pscohen@uri.edu (P.S.C.); 2 Department of Biochemistry and Molecular Biology, Shanxi Medical University, Taiyuan 030001, China; 3 Department of Cell Biology and Medical Genetics, Shanxi Medical University, Taiyuan 030001, China

**Keywords:** biphasic analysis, colorectal cancer, dose reduction index, protein kinase inhibitors, combination targeted therapy

## Abstract

Quantifying the response of cancer cells to a drug, and understanding the mechanistic basis of the response, are the cornerstones for anti-cancer drug discovery. Classical single target-based IC_50_ measurements are inadequate at describing cancer cell responses to targeted drugs. In this study, based on an analysis of targeted inhibition of colorectal cancer cell lines, we develop a new biphasic mathematical model that accurately describes the cell–drug response. The model describes the drug response using three kinetic parameters: ratio of target-specific inhibition, F_1_, potency of target-specific inhibition, K_d1_, and potency of off-target toxicity, K_d2_. Determination of these kinetic parameters also provides a mechanistic basis for predicting effective combination targeted therapy for multi-driver cancer cells. The experiments confirmed that a combination of inhibitors, each blocking a driver pathway and having a distinct target-specific effect, resulted in a potent and synergistic blockade of cell viability, improving potency over mono-agent treatment by one to two orders of magnitude. We further demonstrate that mono-driver cancer cells represent a special scenario in which F_1_ becomes nearly 100%, and the drug response becomes monophasic. Application of this model to the responses of >400 cell lines to kinase inhibitor dasatinib revealed that the ratio of biphasic versus monophasic responses is about 4:1. This study develops a new mathematical model of quantifying cancer cell response to targeted therapy, and suggests a new framework for developing rational combination targeted therapy for colorectal and other multi-driver cancers.

## 1. Introduction

Some cancers rely on a single proliferative driver and its associated signaling pathway. Abl in chronic myeloid leukemia (CML) [1], ErbB2 in some breast cancers [2], and EGFR in some non-small cell lung cancer [3] are a few examples of such mono-driver cancers. Mono-driver cancers can be effectively treated by targeted therapy blocking the function of the proliferative drivers. Small molecule inhibitors or monoclonal antibodies blocking Abl, ErbB2, or EGFR have become the standard of care for these cancers [1,2,3].

Unfortunately, targeted therapy has not been effective for most solid tumors. One key reason for the limited success is that proliferation and viability of most cancers are driven by multiple proliferative drivers, supported by strong genetic evidence [4,5,6]. A recent study of 7664 tumors of 29 cancer types revealed that on average a tumor carries approximately four driver mutations [4]. Some cancers, such as testis and thyroid tumors, carry one driver per tumor, while colorectal cancer and melanoma carry >10 driver mutations per tumor. Mono-agent targeted therapy is ineffective for such multi-driver cancers [7].

A case in point is colorectal cancers (CRC). Treatment for CRC relies mostly on traditional treatment options such as surgery, radiation, and chemotherapy [8,9]. Targeted therapy for CRC has so far narrowly focused on blocking the function of EGFR or angiogenesis (VEGFR) [10], which has not been broadly effective. CRC development is a multi-step process driven by multiple proliferative drivers [4,5,6]. The first event is often a gatekeeping mutation in the adenomatous polyposis coli (APC) gene, which gives the host cell a small growth advantage to slowly develop into a small adenoma. Some cells would acquire additional activating mutations in KRAS, BRAF, PIK3CA, and overexpression of Src and/or other oncogenes, which provide additional proliferative advantages for the full development of a metastatic tumor [6,11,12]. The number of drivers for CRC is estimated to be in the range of three to more than ten [4,6]. Thus, CRC can serve as a model system for developing targeted therapy for multi-driver cancers.

Ample clinical data are consistent with the multi-driver hypothesis for CRC and other cancers. For example, BRAF inhibitors are very effective against melanoma with BRAF V600E/K mutations, but they are not effective for colorectal cancers bearing the same BRAF V600E mutation [13], suggesting that mutated BRAF is not fully responsible for the proliferation of these cancers. Another example is the role of Src kinase in cancer. Despite decades of research demonstrating the crucial role of Src kinases in cancer cell proliferation, survival, adhesion, migration, invasion and metastasis in many tumor types [14,15], Src inhibitors have shown only disappointing therapeutic activity in clinical trials for various solid tumors [16,17]. In fact, not a single Src kinase inhibitor has been approved for targeted therapy. These observations suggest that BRAF or Src is likely to be one of multiple proliferative drivers in these cancers. 

Most efforts of developing combination therapy rely on empirical screening [18,19,20,21], and developing rational approaches to identify effective combination therapy has been a challenge [7,22]. In cases where benefits of combination therapy are reported in clinical trials and in patient-derived xenograft models, most of the benefit is due to patient-to-patient variability without drug additivity or synergy [23]. Thus, there is still an urgent need to develop truly synergistic combinations that are effective on cancers that are refractory to treatment by individual drugs. One of the many barriers to developing effective combination therapy is the lack of appropriate metrics to evaluate cancer cell responses to the targeted drug. Although IC_50_, E_max_ (the maximum effect) and AUC (area under the curve) have all been used to describe cancer cell responses to drugs, they do not accurately reflect the drug response of most cancer cells [24]. Thus, experimentally validated numerical models that capture the complex cell–drug interaction would greatly aid in the formulation of effective combination therapies. 

In this study, we use several multi-driver CRC cell lines and a mono-driver lung cancer cell line to develop a mathematical model for evaluating how multi-driver cancer cells respond to targeted therapy. The model breaks down cell response to targeted drugs into three mechanism-based kinetic parameters, that provide a foundation for identifying mechanism-based and highly synergistic drug combinations for multi-driver cancer cells. 

## 2. Results

### 2.1. Viability of HT-29 Cells Is Sensitive to Four Protein Kinase Inhibitors

HT-29 cells have been widely used to study mechanisms of colorectal cancer cell proliferation and for drug discovery [25]. Genetic and biochemical characterizations suggest that this cell line is a good model for multi-driver proliferation. The HT-29 genome contains 676 coding mutations, including 24 mutations in 21 cancer census genes, such as two mutations in APC, two mutations in BRAF, and one mutation each in PIK3CA, SMAD4, and TP53 [26]. Associations of Src [27], BRAF [28], PI 3-kinase pathway [29] and IGF-1R [30] with the proliferation of HT-29 have been reported. We evaluated the inhibition of HT-29 viability by 18 protein kinase inhibitors that collectively block most oncogenically important kinases, including receptor and soluble PTKs and protein kinases in the MAP kinase and PI 3-kinase pathways [31,32]. To quantitatively understand the dynamic and complex response of cancer cells to targeted therapy, we determined the dose responses at 16 concentrations from 0.6 nM to 20 µM. Of the 18 kinase inhibitors, HT-29 cells were moderately sensitive to four: HG6-64-1 (IC_50_ = 0.243 ± 0.02 µM), dasatinib (IC_50_ = 0.403 ± 0.06 µM), BMS-754807 (IC_50_ = 0.99 ± 0.09 µM), and AZD-6244 (IC_50_ = 1.334 ± 0.18 µM) (Table 1). No other inhibitors had IC_50_s below 8 µM, and about half of the inhibitors did not reach 50% inhibition at 20 µM. The IC_50_ values reported in Table 1 were derived by graphically identifying the concentration that corresponded to 50% inhibition.

HG6-64-1 is a specific BRAF inhibitor [33] and AZD-6244 (selumetinib) is a Mek kinase inhibitor [34]. The HT-29 genome contains two BRAF mutations, V600E and T119S [26]. The V600E mutation activates BRAF, which phosphorylates and activates Mek. The inhibition of HT-29 viability by these two inhibitors suggests that the BRAF-activated MAP kinase pathway is a driver for HT-29 proliferation and/or viability. Dasatinib is a broad inhibitor for kinases in the Abl, Src, and PDGFR families [35]. Due to the fact that the cells did not respond to the Abl inhibitor imatinib, or the PDGFR inhibitor sunitinib, and Src is over-expressed in HT-29 cells with a Z score of 2.99 [26] and activated [27], the most likely dasatinib target is Src. BMS-754807 is an inhibitor for the insulin receptor (IR) and the insulin-like growth factor 1 receptor (IGF-1R) [36]. These results suggest that BRAF/MAP kinases, Src kinases and IR/IGF-1R kinases all contribute to the viability of HT-29 cells. 

### 2.2. IC_50_ Values Do Not Accurately Reflect How HT-29 Cells Respond to Targeted Therapy

Traditionally, cell response to a drug is measured by IC_50_, the concentration that inhibits cell viability by 50%, which is commonly calculated by fitting the data into a single target inhibition equation. However, the HT-29 response data clearly and consistently did not fit the single target binding equation I = [D]/([D] + IC_50_), where I is the relative inhibition, and [D] is the drug concentration (Figure S1A–D). The dashed lines in Figure S1 were the best fitting single target kinetic curves for HG6-64-1, BMS-754807, dasatinib and AZD-6244. The poor fitting is evident judged visually or by the root mean square error (RMSE), and is clearly not the result of experimental inaccuracy. The kinetically best fitting IC_50_ values were often significantly different from the visually determined IC_50_ values reported in Table 1. 

In an attempt to better understand the dose responses, the data were also fitted to a modified Hill equation: I = I_max_ × D^n^/(IC_50_*^n^ + D^n^). In this equation, the relative inhibition (I) is expressed as a function of drug concentration (D) determined by three parameters: I_max_, IC_50_* and n. I_max_ is the maximal relative inhibition a drug can achieve at saturating concentration, IC_50_* is the drug concentration that achieves half of maximal inhibition, and n is the Hill coefficient, or slope. We did not use the fourth parameter of the full Hill equation, I_0_ (inhibition in the absence of an inhibitor), because there is no inhibition at the drug concentration of zero. Fitting the data into the above equation produced the graphs shown in Figure S1. 

The equation fit the data reasonably well, producing IC_50_* values that reflected how potently the drugs inhibited cell viability. However, each drug generated a highly variable n value significantly less than 1 (ranging from 0.34 for BMS-754807 to 0.84 for HG6-64-1), suggesting varying levels of negative cooperativity. As there is no obvious explanation for such apparent negative cooperativity, it is presumed to reflect some undefined biological property of the system. Such a mathematically accommodating, but biologically undefined, parameter is considered one of the limitations of the Hill equation when it is applied to drug dose response curves.

**Figure 1:**
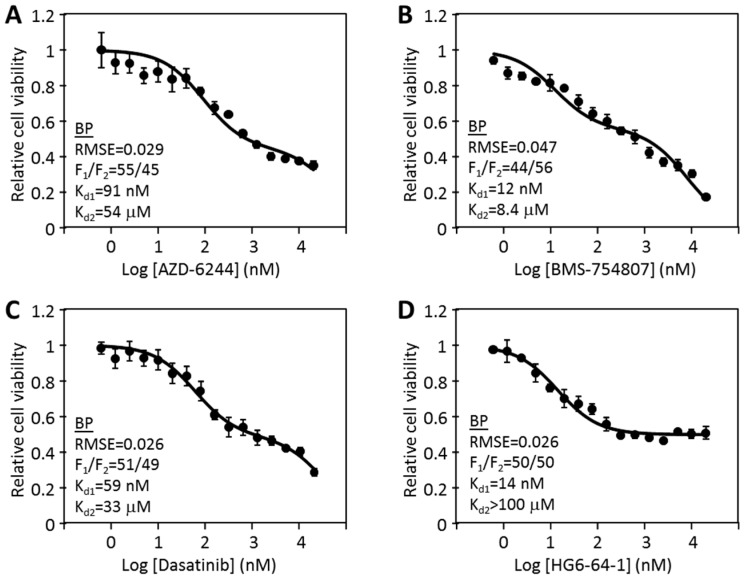
Biphasic analyses of the inhibition of HT-29 cells by protein kinase inhibitors. The dose responses data of HT-29 cells to AZD-6244 (**A**), BMS-754807 (**B**), dasatinib (**C**), and HG6-64-1 (**D**) were curve-fitted to the biphasic inhibition equation I = F_1_ × [D]/([D] + K_d1_) + F_2_ × [D]/([D] + K_d2_). Each set of inhibition data represents two independent experiments in triplicate, with standard errors presented as error bars. The inhibitory parameters obtained from curve fitting are presented in the graphs.

Furthermore, three of the four drugs also produced an I_max_ that is significantly less than 1, suggesting that even when a drug saturates its target, it still does not fully inhibit cell viability. This indicated that the target kinase of the drug is only partially responsible for cell viability. We further noticed that some drugs, such as dasatinib and BMS-754807, displayed a biphasic inhibition pattern, with a nM inhibitory phase and µM inhibitory phase. The nM inhibition was assumed to correlate to the target-specific inhibition, while the µM inhibition likely reflected an off-target effect. Based on these considerations, we decided to further modify the Hill equation to set n at 1, and add a second phase inhibition. Thus the equation was modified into a biphasic equation: I = F_1_ × [D]/([D] + K_d1_) + F_2_ × [D]/([D] + K_d2_). In this equation, the inhibition (I) has two phases: F_1_ and F_2_ as fractions of total cell viability with their respective K_d_s (K_d1_ and K_d2_). The biphasic assumption dictates that F_1_ + F_2_ = 1 (or 100%), thus F_2_ is not an independent variable, but a derivative of F_1_. The biphasic curve fitting graphs are shown in Figure 1A–D. In every case, the biphasic equation fits the data well, judged visually or by the RMSE. The RMSE values for the biphasic equation fitting were often 5–10 times lower than the monophasic equation fitting, and similar to those of the full Hill equation fitting.

The biphasic kinetic curve fitting revealed the best fitting F_1_, F_2_, K_d1_ and K_d2_ for each drug. The inhibition by HG6-64-1 had an F_1_ of 57% with a K_d1_ of 19.8 nM, and an F_2_ of 43% with K_d2_ > 1000 µM (largely resistant). This analysis indicated that HG6-64-1 inhibited 57% of cell viability with a K_d_ of 19.8 nM, but left the remaining portion of viability unaffected. Similarly, the responses to the other drugs also consisted of an F_1_ with a low nM K_d1_ and an F_2_ with a >10 µM K_d2_. These analyses suggest that the cell responses can be accurately described by three constants: F_1_/F_2_ ratio, K_d1_ and K_d2_ in the biphasic model.

### 2.3. Biological Meaning of Biphasic Inhibition

**Figure 2:**
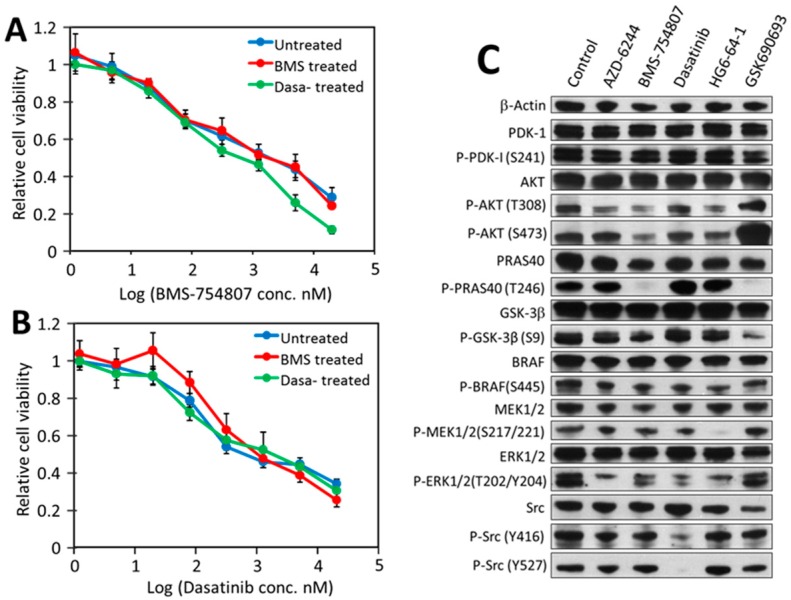
Effects of drug treatment on HT-29 drug sensitivity and signaling. HT-29 cells were pre-treated with 2 µM BMS-754807, 2 µM dasatinib, or no drug for 72 h. The residual cells were recovered from each treatment and tested for sensitivity to BMS-754807 (**A**) and dasatinib (**B**). (**C**) Effects of protein kinase inhibitors on the status of key signaling pathways. The results with standard errors were from three independent experiments in triplicate. HT-29 cells at 70% confluency were treated with no drug (control), 5 µM AZD-6244, 2 µM BMS-754807, 1 µM dasatinib, 0.12 µM HG6-64-1 or 5 µM GSK690693 for 2 h. The proteins in the treated cells were fractionated by SDS-PAGE and probed by different antibodies against the indicated proteins. Protein sample loadings were equalized based on the level of β-actin. Full pictures of the Western blots and the densitometry scans are presented in Figures S2 and S3.

Two plausible scenarios are consistent with the biphasic inhibition patterns. One is that HT-29 cells contain two subpopulations, one being more sensitive (F_1_ and K_d1_) than the other (F_2_ and K_d2_). If this scenario is true, treatment with a drug should eliminate/reduce the subpopulation sensitive to the drug. To test this scenario, the HT-29 cells were treated with 2 µM dasatinib, BMS-754807, or no drug for three days, and the residual cells were recovered and retested for their sensitivity toward the two drugs. The recovered cells from the three treatments displayed virtually identical sensitivity to either dasatinib or BMS-754807 (Figure 2A,B). This result argues against the two-population possibility. 

The second scenario is that each drug causes the F_1_ inhibition by blocking a high affinity driver and causes the F_2_ inhibition through a non-specific off-target mechanism. This scenario is consistent with two basic features of this experimental system: (1) the kinase inhibitors used inhibit their targets with low nM K_d_s, but also inhibit other protein kinases non-specifically at µM concentrations; and (2) HT-29 cells are sensitive to multiple kinase inhibitors, suggestive of multiple independent drivers. The combination of these two features makes it possible that each drug partially blocks viability when it inhibits a high affinity target, and inhibits the remaining portion of viability when the drug concentration reaches a cytotoxic level. It is well established that most kinase inhibitors cause cytotoxicity at µM concentrations. Elimination of the first scenario leaves the second as the most plausible explanation. 

### 2.4. Src, IR/IGF-1R/AKT Signaling and the MAP Kinase Pathway Are All Partially Responsible for Driving HT-29 Cell Proliferation and Viability

To verify that AZD-6244, BMS-754807, dasatinib, and HG6-64-1 are indeed blocking distinct drivers, we determined the effects of each drug on the activation status of key signaling proteins, including insulin receptor (IR), IGF-1R, PDK1, AKT, AKT substrates PRAS40 and GSK3β, BRAF, Mek, Erk and Src (Figure 2C). For this analysis, we also included an AKT inhibitor, GSK690693 [37], even though GSK690693 surprisingly did not inhibit HT-29 viability (Table 1). 

The immunoblots in Figure 2C revealed the following. (1) AZD-6244 (Mek inhibitor) strongly inhibited the phosphorylation of Erk, a Mek substrate. It may have also inhibited, to a lesser degree, the activation of AKT. (2) Western blots of the phosphorylated IR and IGF-1R were not informative, due to the detection of a large number of protein bands smaller than the expected sizes of these two receptors (Figure S4). Despite repeated attempts under different conditions in consultation with the antibody manufacturer, these lower bands, either degradation products or nonspecific recognition, persisted. However, BMS-754807 (inhibitor of IR and IGF-1R) clearly inhibited Akt phosphorylation on both T308 and S473, an indication of Akt activation [38]. The blockade of Akt activation was confirmed by the undetectable phosphorylation of PRAS40 and reduced phosphorylation level of GSK3β, two direct substrates of Akt [38]. In addition, the phosphorylation level of BRAF, MEK and ERK also decreased in response to BMS-754807. These results indicated that blocking IR/IGF-1R by BMS-754807 resulted primarily in the down-regulation of AKT, but also the MAP kinase pathway. (3) Dasatinib completely blocked Src phosphorylation on Y527 by another PTK, Csk [39], and significantly inhibited Src autophosphorylation on Y416. This is consistent with earlier reports that both Src and Csk are sensitive to dasatinib [40]. Dasatinib also appeared to partially inhibit Mek and Erk activation, suggestive of crosstalk between Src and the MAP kinase pathway. (4) HG6-64-1 [33] (a BRAF inhibitor) moderately inhibited BRAF autophosphorylation, but completely blocked Mek phosphorylation by BRAF, and reduced Erk phosphorylation by Mek. It also reduced the phosphorylation of AKT and PRAS40. These results suggested considerable cross talk between the MAP kinase, IR/IGF-1R/Akt and Src pathways. 

GSK690693 is a specific Akt inhibitor [37]. Although it did not inhibit HT-29 cell viability (Table 1), it completely blocked the phosphorylation of PRAS40 and strongly inhibited phosphorylation of GSK3β (Figure 2C), confirming its ability to block Akt kinase function. However, it also strongly activated Akt phosphorylation on S473, as previously reported [37,41,42]. Phosphorylation of Akt on S473 is catalyzed by PDK2, a kinase activity attributed to several different kinases [43], especially rapamycin complex 2 (mTORC2) [44]. Activation of Akt S473 phosphorylation by GSK690693 was thought to be a feedback mechanism by mTORC2. mTORC2 also phosphorylates other AGC kinases at their corresponding hydrophobic motif residues [43]. The activation of PDK2/mTORC2 by GSK690693 may explain its lack of inhibition of HT-29 viability. 

The above results overall demonstrated that AZD-6244, BMS-754807, dasatinib, and HG6-64-1 indeed blocked their respective target kinases, and caused distinct patterns of signaling inhibition. These results further support the multi-driver proliferation hypothesis in HT-29 cells.

### 2.5. HT-29 Cell Viability Can Be Effectively and Synergistically Blocked by Combinations of Drugs, Each Blocking an Independent Driver 

**Figure 3:**
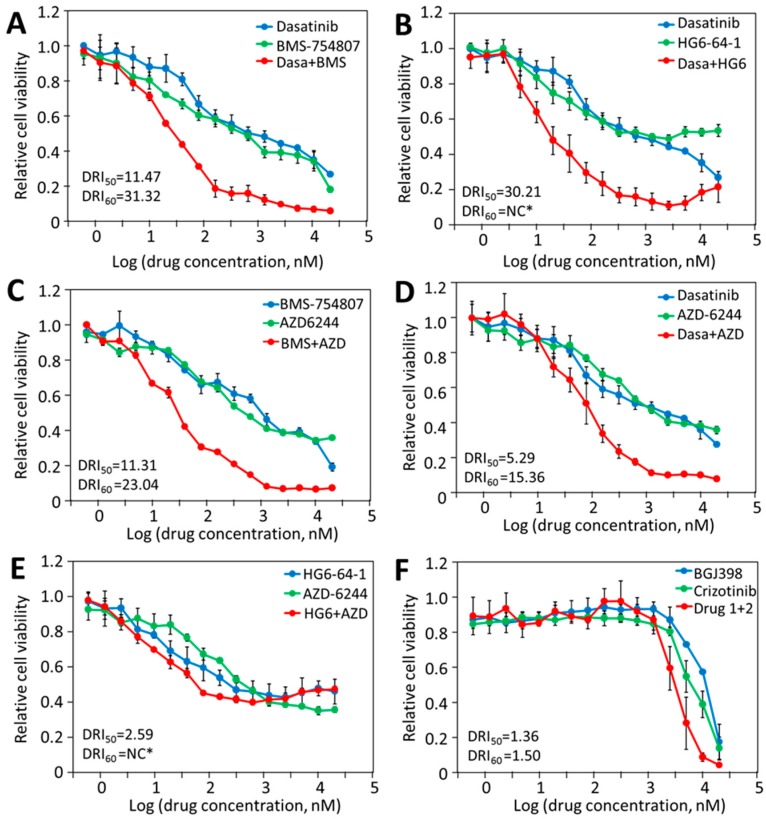
Synergistic inhibition by drugs blocking independent protein kinases. The responses of HT-29 cells to different drug combinations were compared to those to the individual drugs. (**A**), dasatinib and BMS-754807; (**B**), dasatinib and HG6-64-1; (**C**), AZD-6244 and BMS-754807; (**D**), dasatinib and AZD-6244; (**E**), HG6-64-1 and AZD-6244; (**F**), BGJ398 and crizotinib. The results are representative of two independent experiments. The dose reduction index (DRI) values at 50% and 60% inhibition are presented. * NC, not calculated because one or both of the drugs did not reach 60% inhibition at 20 µM.

If the multi-driver proliferation mechanism is true, then the combination of inhibitors with non-overlapping F_1_ targets should synergistically block the proliferation. The effects of pair-wise drug combinations (1:1 ratio) on HT-29 viability were determined. In Figure 3A, the IC_50_s for dasatinib and BMS-754807 were 500 nM and 790 nM, respectively, and the IC_60_s for either drug was above 2 µM. The IC_50_ and IC_60_ for the drug combination were 28 nM and 50 nM, respectively. The dose reduction index (DRI), a measure of how many folds the drug doses may be reduced by the combination to achieve a given inhibition level [45], was 11-fold at 50% inhibition and 31-fold at 60% inhibition (Figure 3A), meaning that the combination is 31 times more effective than the two drugs alone in achieving 60% inhibition. The combinations of dasatinib with AZD-6244 or HG6-64-1, and the combination of BMS-754807 with AZD-6244, also showed dramatic synergy (Figure 3B–D). These results suggest that the drugs in these pairs (dasatinib/BMS-754807, dasatinib/HG6-64-1, dasatinib/AZD-6244, and BMS-754807/AZD-6244) exert their effects on HT-29 cells largely independently, which is consistent with the multi-driver proliferation hypothesis.

We also tested the combination of HG6-64-1 and AZD-6244, inhibitors of two consecutive steps in the MAP kinase pathway. As their F_1_ effects on HT-29 cell viability would largely overlap, their combination would be redundant rather than complementary/synergistic. The combination indeed produced very little synergistic benefit (Figure 3E), still retaining approximately 40% viability when both drugs were at 20 µM. Thus, the portion of viability that is resistant to HG6-64-1 is also largely resistant to AZD-6244, and independent of the MAP kinase pathway. 

Two drugs, BGJ398 (FGFR inhibitor) [46] and crizotinib (Met inhibitor) [47] did not inhibit HT-29 viability in the nM range, but inhibited HT-29 viability at around 10 µM (Table 1, Figure 3F), likely through off-target toxicity. The combination of these two drugs did not result in synergistic inhibition (Figure 3F), indicating that only target-specific effects by drugs on different targets would have synergistic effects.

The combination of three drugs, dasatinib, BMS-754807 and AZD-6244, had an IC_50_ of 30 ± 5 nM on HT-29 cells, marginally lower than the IC_50_s of the pair-wise combinations, suggesting that blocking any two of the three pathways is largely sufficient in blocking HT-29 viability. This overlap between the effects of these drugs is consistent with the cross-talk observed in the Western blots (Figure 2C).

### 2.6. Other CRC Cancer Cell Lines Follow a Similar Pattern in Their Responses to Individual Kinase Inhibitors and Inhibitor Combinations 

**Figure 4:**
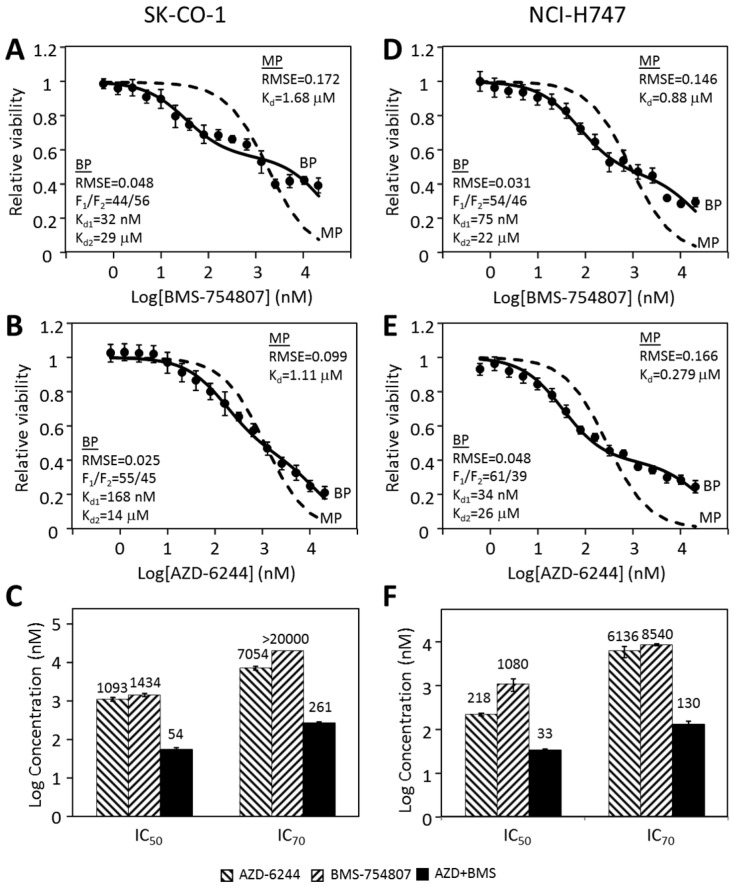
Proliferative inhibition of CRC cell lines SK-CO-1 and NCI-H747. The inhibition of SK-CO-1 (**A**,**B**) and NCI-H747 (**D**,**E**) cells by BMS-754807 (**A**,**D**) and AZD-6244 (**B**,**E**) was analyzed by the monophasic and biphasic kinetic equation. The IC_50_ and IC_70_ values for AZD-6244, BMS-754807 and their combination are presented for SK-CO-1 (**C**) and NCI-H747 (**F**). The data presented were from six sets of data from two independent experiments consisting in triplicate. The average IC_50_ and IC_70_ values in nM were presented above each bar graph.

We previously reported [31] that the CRC cell lines SK-CO-1 and NCI-H747 were mildly inhibited by both BMS-754807 and AZD-6244 and much more potently by the two inhibitors combined. We tested if the responses of these two cells to BMS-754807 and AZD-6244 were also biphasic (Figure 4). The responses of SK-CO-1 to BMS-754807 and AZD-6244 clearly did not fit the monophasic equation (Figure 4A,B) (dashed lines), indicated by a poor fitting curve with high RMSEs. Biphasic analyses of the same data (Figure 4A,B, solid lines) yielded much better fitting curves. The two drugs had F_1_ values of 44% and 55% respectively, and K_d1_s 32 nM and 168 nM, respectively. This suggested that the targets of BMS-754807 and AZD-6244 are respectively responsible for 44% and 55% of SK-CO-1 viability. The results suggested that the MAP kinase pathway and the BMS-754807-sensitive pathway, the IR/IGF-1R initiated Akt signaling pathway, each counted for approximately half of SK-CO-1 viability.

**Figure 5:**
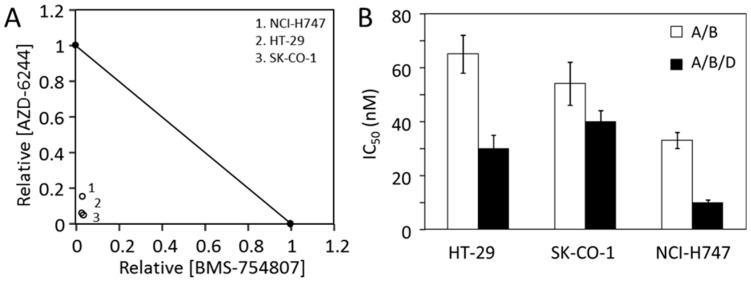
Potency of CRC cell inhibition by the drug combinations. (**A**) The dramatic synergy of BMS-754807 and AZD-6244 is demonstrated by a relative isobologram. The IC_50_s of all three cell lines for BMS-754807 and AZD-6244 and the two drug combination are normalized by the individual drug IC_50_s. (**B**) Summary of the IC_50_ values of AZD-6244/BMS-754807 combination (A/B) and AZD-6244/BMS-754807/dasatinib triple combination (A/B/D) for all three CRC cell lines. Averages from six sets of data with standard error are presented.

As shown in 4C, SK-CO-1 cells were much more potently inhibited by the combination of AZD-6244 and BMS-754807 than either drug alone. The IC_50_ and IC_70_ were more than an order of magnitude lower than the corresponding values for either drug alone. The synergy was especially pronounced at higher inhibition levels. The IC_70_ values were >7 µM for either drug alone, but only 261 nM for the drug combination. Very similar biphasic inhibitory patterns and synergistic inhibition were observed for NCI-H747 cells (Figure 4D–F). These results suggest that multi-driver proliferation is a common mechanism in these cells and combination targeted therapy may be broadly effective for CRC. The synergy and potency of double and triple drug combinations for three CRC cell lines are summarized in Figure 5A,B.

### 2.7. Mono-Driver Cancer Cell Responses to Targeted Therapy Is Monophasic

To validate that the biphasic responses and combination synergy are unique to multi-driver cancer cells, we determined how a mono-driver cancer system would behave in these analyses. Targeted therapy using EGFR inhibitors has become the standard of care for non-small cell lung cancers that contain activating EGFR mutations [3], even though not all patients with EGFR mutations respond to EGFR inhibitors, such as erlotinib [48]. HCC-827 is a model cell line for such cancers [49]. It contains four mutations that activate EGFR, which drives the proliferation [26]. HCC-827 cells are indeed very sensitive to several EGFR inhibitors, gefitinib, afatinib and erlotinib, with IC_50_s between 7.8 nM and 15.4 nM (Table 2). They are also inhibited by EGFR inhibitors neratinib and lapatinib, although with higher IC_50_s. The cells are also sensitive to dasatinib and bosutinib with IC_50_s of 140 and 273 nM, respectively. 

**Figure 6:**
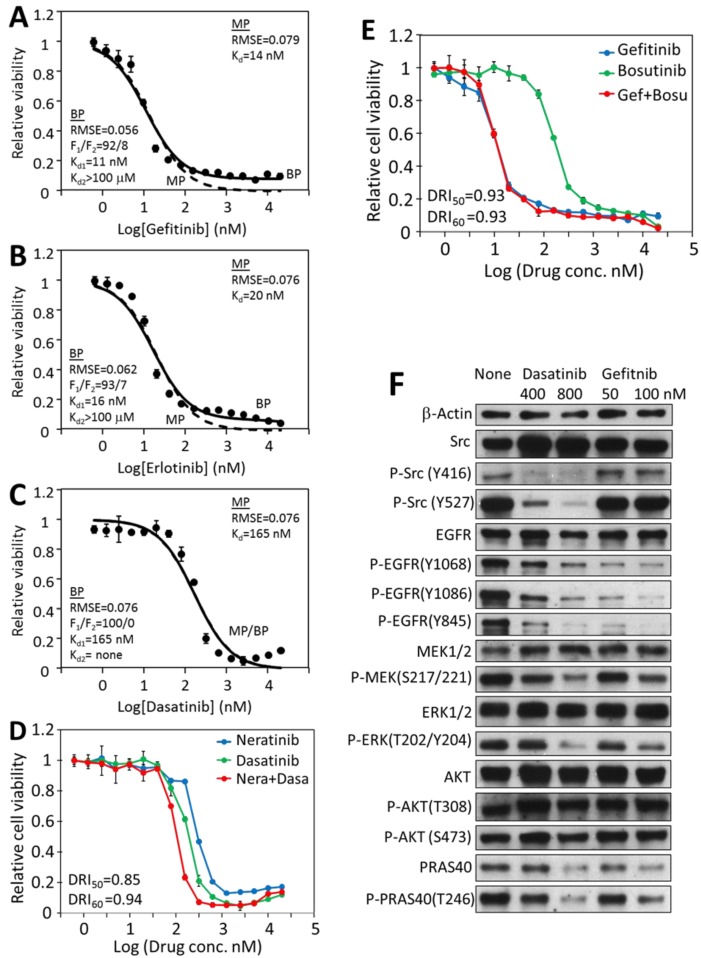
Kinetic and signaling analyses of the responses of HCC-827 lung cancer cells to EGFR and Src kinase inhibitors. Monophasic and biphasic analysis of HCC-827 inhibition by EGFR inhibitor gefitinib (**A**), erlotinib (**B**) and Src inhibitor dasatinib (**C**). The biphasic curve (solid line) and the monophasic curve (dashed line) in Figure 6C completely matched each other, as F1 was 100%. (**D**), Combination analyses of dasatinib and neratinib on HCC-827 viability. (**E**) Combination analysis of gefitinib and bosutinib on HCC-827 viability. (**F**), Western blot analysis of the effects of dasatinib and gefitinib on the protein and phosphorylation levels of selected signaling proteins. The analyses were carried out as described for HT-29 cells. Full pictures of the Western blots and the densitometry scans are presented in Figures S5 and S6.

In contrast to the CRC cells, the HCC-827 responses to EGFR and Src inhibitors followed single-target equation. For example, the gefitinib inhibition data fit well to the monophasic equation, with an IC_50_ of 14.1 nM (Figure 6A, dashed line). Fitting the same data to the biphasic equation resulted in a very similar graph with an F_1_ of 92%, and a K_d1_ of 11.2 nM (Figure 6A, solid line), indicating that the target-specific F_1_ response is predominantly responsible for gefitinib inhibition of HCC-827. Similar results were obtained for erlotinib inhibition (IC_50_ = 19.6 nM, F_1_ = 93%, K_d1_ = 16.1 nM) (Figure 6B) and dasatinib (IC_50_ = 165 nM, F_1_ = 100%, K_d1_ = 165 nM) (Figure 6C). These results indicated that any of these drugs inhibiting its specific target (EGFR or Src) alone was largely sufficient to block HCC-827 viability. The fact that inhibiting either EGFR or Src would be sufficient to block the viability indicates that EGFR and Src are dependent on each other in supporting the viability of this cell line. Therefore, EGFR and Src are not independent drivers for HCC-827 proliferation and viability. 

At µM concentrations, dasatinib caused a slight but consistent uptick in HCC-827 cell viability (Figure 6C). This is likely a reflection of the complex effects of dasatinib on cell signaling and biochemistry. As a broad spectrum kinase inhibitor, it is conceivable that it could directly or indirectly stimulate cell viability by inhibiting some kinases. It has been previously reported that dasatinib induced elevated levels of phosphorylated AKT protein as well as ERK1/2 proteins in FaDu and A431 cells [50], and the activation of these growth stimulating kinases could result in the stimulation of cell viability. Whether or not the apparent stimulation of HCC-827 cells is due to this mechanism is yet to be determined.

The synergy between Src and EGFR inhibitors on HCC-827 was also tested (Figure 6D,E). Neither the neratinib/dasatinib combination nor the gefitinib/bosutinib combination displayed significant synergy, indicating that blocking EGFR, Src or both achieved largely the same effect on HCC-827 viability, further demonstrating that Src and EGFR are not independent drivers in HCC-827. 

A Western blot analysis demonstrated that (Figure 6F) gefitinib blocked EGFR phosphorylation and activation, and the EGFR downstream pathways, such as the phosphorylation of Mek, Erk, and Akt substrate PRAS40. The gefitnib treatment did not affect the Src phosphorylation on Tyr416 or Tyr527. Dasatinib not only blocked Src phosphorylation on Tyr416 and its activation, but also blocked EGFR phosphorylation, Mer, Erk and PRAS40 phosphorylation. Therefore, blocking Src function also prevented EGFR signaling, indicating that the function of EGFR in activating the MAP kinase pathway and the Akt pathway is dependent on Src function. This finding is consistent with reports that EGFR activation is dependent on recruiting Src to phosphorylate several phosphorylation sites on EGFR [51,52]. These results suggest that mutation-activated EGFR is the true driver of HCC-827 proliferation, while Src is an essential component in the EGFR-initiated signaling pathways. 

### 2.8. Inhibition of Cancer Cell Viability by Dasatinib Is Mostly Biphasic

Even though the biphasic inhibition pattern is strongly supported in our hands, such an inhibitory pattern has not been reported in the literature to our knowledge. The only report [53] that comes close to it is that 28% of cancer cell–drug response curves were better described by a multi-phasic model. To determine if the biphasic inhibition model is broadly applicable, we applied the analysis to dasatinib dose response data that are available in the Genomics of Drug Sensitivity in Cancer database [54].

**Figure 7:**
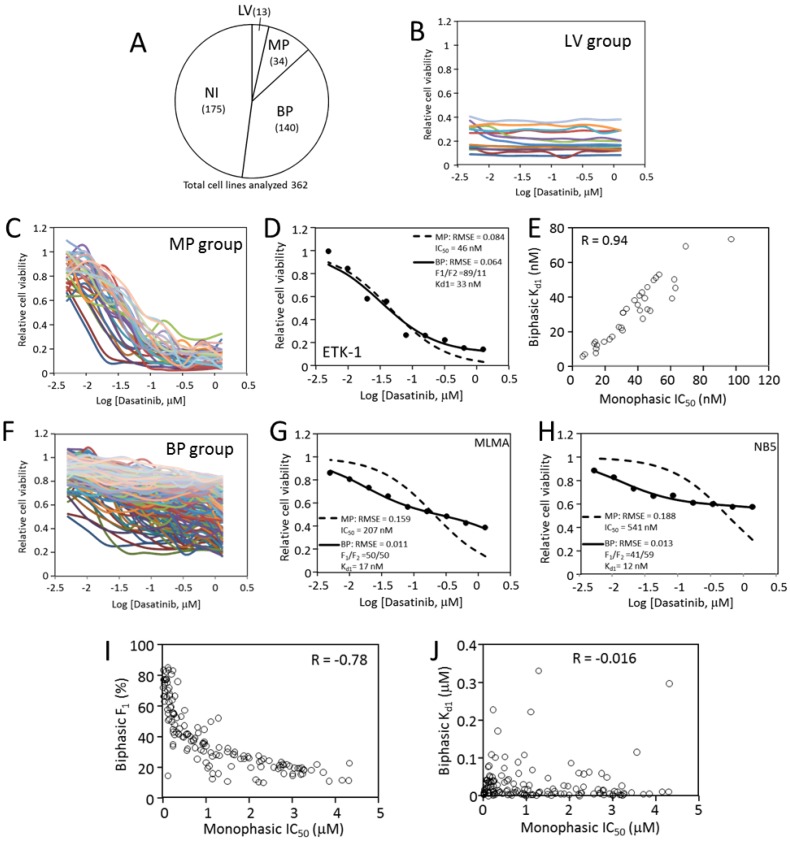
Analysis of dasatinib inhibition of cancer cells. (**A**) Four categories of cancer cell responses to dasatinib and the number of cell lines in each (Total = 362). LV, low viability; MP, monophasic response; BP: biphasic response; NI, no inhibition. See main text for details about each category. (**B**) Dose responses to dasatinib by low viability (LV) cells. (**C**) Dose responses to dasatinib by monophasic response (MP) cells. (**D**) Monophasic and biphasic analyses of ETK-1 cell line to dasatinib as an MP example. (**E**) Positive correlation between monophasic IC_50_ and K_d1_ in biphasic analysis for MP cells. (**F**) Dose responses to dasatinib by biphasic response (BP) cells. (**G**) Monophasic and biphasic analyses of MLMA cell line to dasatinib as a BP example. (**H**) Monophasic and biphasic analyses of NB5 cell line to dasatinib as a BP example. (**I**) Inverse correlation between monophasic IC_50_ and F_1_ in a biphasic analysis for BP cells. (**J**) Lack of correlation between monophasic IC_50_ and K_d1_ in a biphasic analysis for BP cells.

In this dataset, the responses of 426 cancer cell lines to dasatinib at nine concentrations from 5 nM to 1.28 µM (2× dilution series) were determined. The data were collected through a high throughput screen, with each cell line/drug concentration tested in one well in the 384-well format [54]. We removed dose responses that contained obvious scattering, and the remaining 362 datasets were analyzed. The responses to dasatinib were divided into four categories (Figure 7A): Category LV (low viability) contains 13 cell lines characterized by low viability readings regardless of the drug concentration (Figure 7B). All 13 cell lines are suspension blood cancer cell lines. The second category (category NI, no inhibition) contains 175 cells, and they are not inhibited by dasatinib up to 1.28 µM. Due to the lack of concentration-dependent inhibition, both of these categories were not analyzed further for this study.

The remaining 174 cell lines responded to dasatinib in dose-dependent manners with varying levels of sensitivity. Both monophasic and biphasic analyses were performed on these cell lines (Data S1). In the biphasic analysis, the F_1_ value varied from 100% to about 10%. Thirty-four (34/174) cell lines had an F_1_ above 85%, and these cells were inhibited more than ~80% at 1.28 µM dasatinib (Figure 7C). Their responses fit the monophasic equation and the biphasic equation similarly well, with comparable RMSE values (0.086 + 0.004 for MP analysis versus 0.074 + 0.004 for BP analysis). Kinetic analyses of the ETK-1 cell line are shown as an example of this group in Figure 7D. In this group, the K_d1_ values closely aligned to the IC_50_ values from the monophasic analysis (Figure 7E). This group is called the MP category, where inhibiting the dasatinib target is largely sufficient to block cell viability. Cells in this category are likely fully or nearly fully dependent on a proliferative driver that is sensitive to dasatinib, such as abl, Src of PDGFR family kinases.

The remaining 140 cell lines (BP category) display clear biphasic characteristics with F1 < 85%. They all have shallow response curves, displaying clear inhibition at low dasatinib concentrations, but still retain considerable cell viability even at 1.28 µM (Figure 7F). These response datasets fit the biphasic equation much better than the monophasic equation (RMSE = 0.142 ± 0.004 for MP analysis, and 0.038 ± 0.002 for BP analysis). Two examples of the monophasic and biphasic fitting comparisons are shown in Figure 7G,H. As a group, the IC_50_ values for these cells range from ~100 nM to ~4 µM, and the increase in IC_50_ values inversely correlates to the decrease in F_1_ (Figure 7I). Because the highest dasatinib concentration used in the screen was 1.28 µM, too low to cause significant off-target toxicity, the data do not yield a reliable K_d2_. It is also interesting to note that the K_d1_ for this group mostly remained below 100 nM, even though the IC_50_ are up to ~4 µM (Figure 7J), indicating that the affinity between dasatinib and its target is largely independent of the cell host. The ratio of monophasic versus biphasic responses is 34:140, suggesting that most cancer cells are likely to be multi-driver dependent. This analysis indicates that even though the biphasic response pattern to targeted therapy has not been widely appreciated, it is actually very common.

## 3. Discussion

Most cancers rely on multiple drivers for full proliferation, and require drug combinations for effective targeted therapy. Currently, most drug combinations are discovered by empirical testing [18,19], a rational and a mechanism-based platform would greatly aid in the formulation of effective drug combinations. In the current study, we investigated how multi-driver cancer cells respond to targeted therapy, and developed a mathematical model that quantitatively describes the response of multi-driver cancer cells to targeted therapy. This analysis suggested that multi-driver cancer cells have a biphasic response to targeted agents: one caused by the drug blocking a high affinity target, and the other due to off-target toxicity. The target-specific response correlates to the role a drug target plays in the proliferation of a cancer cell. Quantifying the target-specific effects of a given drug on cancer cells provides a mechanistic basis for a rational approach for identifying effective drug combinations for multi-driver cancers. We demonstrated that combinations of drugs, each blocking a specific driver, potently and synergistically block the viability of cancer cells that are refractory to inhibition by individual drugs.

### 3.1. Multi-Driver Cancers Are a Major Challenge to Targeted Therapy

Despite the dramatic successes targeted cancer therapy has enjoyed, it faces two major challenges: acquired resistance [55,56] and lack of sensitivity. A mono-driver cancer can be effectively treated initially with targeted therapy, but it acquires resistance to the same treatment after the initial sensitive period. The acquired resistance is due to the selection for cells that contain resistance-conferring mutations in the original driver, or new alternative signaling pathways being activated [57,58]. The acquired resistance can be overcome by developing drugs that can block the mutated driver or newly activated driver. Combination therapy that targets more than one step in a proliferative signaling pathway also helps prevent the development of acquired resistance. The second challenge is that most cancers are naturally refractory to targeted therapy. The lack of sensitive response suggests that a cancer is not addicted to a predominant driver or pathway, and multiple proliferative drivers are sustaining the proliferation. This is consistent with genetic evidence that most cancer cells contain multiple driver genes and driver mutations [4,6]. It is logical that only a combination of drugs, each blocking an independent driver, would be effective in stopping multi-driver cancers.

Despite considerable preclinical and clinical effort at developing combination therapy, effective combination cancer therapy is still elusive [7,23]. A recent analysis of many FDA-approved drug combinations revealed that most of the benefits are derived from patient-to-patient variability without drug additivity or synergy [23]. This was also found to be true for combinations tested in patient-derived tumor xenografts [23]. Thus, how to develop effective combination therapy to treat multi-driver cancers that are naturally insensitive to individual drugs is still a challenge. Underlying that challenge is the fact that how multi-driver proliferation responds to targeted therapy is still poorly understood [6]. 

Multi-driver proliferation can account for many of the existing challenges in targeted therapy. For example, Src kinases are shown to play important roles in the proliferation of various cancers [14,15], but clinical trials of Src inhibitors for such cancers have produced disappointing results [16,17]. The discrepancy between cell-based studies and clinical trials has been a major enigma in targeted therapy. Another example is the BRAF V600E mutant which is found in many cancer types. However, targeting BRAF is an effective treatment in melanoma [13], but not for colorectal cancers containing the same mutation [13,59]. A plausible explanation is that Src or BRAF are likely one of the multi-drivers in some cancers. Even though Src kinases are associated with many cancer types, they have not been shown to be an exclusive driver in any cancer. As we demonstrated in this study, BRAF V600E and Src are part of the multi-driver mechanism in the HT-29 cancer cell line. Furthermore, in most of the dasatinib-sensitive cancer cells, the dasatinib target, likely Src in many cases, is one of the drivers in multi-driver cells. For such multi-driver cancers, finding effective drug combinations to stop most, if not all proliferative drivers, is needed for effective combination targeted therapy.

### 3.2. Multi-Driver Proliferation Challenges Traditional Pharmacological Metrics for Analyzing Cancer Cell–Drug Response 

Traditionally, the effect of a cancer drug on cancer cells is measured by the IC_50_, which works well for traditional drugs, and measures how potently a drug kills cancer cells. As previously noted [24] and further demonstrated in the current study, IC_50_ does not capture how a targeted therapy agent affects a multi-driver cancer cell. Current mathematic models for calculating IC_50_ are derivatives of the Hill equation which was developed to describe O_2_ binding to hemoglobin. They are all based on the assumption of cooperative binding of a ligand to a single target. Two assumptions of the Hill equation are not consistent with the response of multi-driver cancer cells to targeted therapy. First, there is no evidence that kinase inhibitors bind to kinase with cooperativity. Second, kinase inhibitors tend to inhibit many kinase targets that are playing various roles in regulating cell viability and proliferation, thus inhibition of multi-driver cancer cells by targeted therapy is clearly a multi-target system. These considerations are clearly reflected in the dose response data of colorectal cancer cells. An analysis of the response of multi-driver cancer cells to kinase-based targeted therapy resulted in the evolution of the Hill equation into a biphasic mathematical model. Using HT-29 and other CRC cell lines, we demonstrated that multi-driver cancer cells respond to targeted therapy blocking one driver in two phases: a target-specific phase and an off-target toxicity phase. The biphasic response can be represented by three parameters, F_1_/F_2_ ratio, K_d1_ and K_d2_. Each parameter has a distinct mechanistic basis: F_1_/F_2_ ratio represents how much a role a driver may play in the cell proliferation and viability; K_d1_ represents the affinity a drug has for the driver; and K_d2_ represents the potency of a drug’s general toxicity to a host cell. The three-parameter mathematical model accurately describes the drug response of all the cancer cells we have examined so far. If a cancer cell is mono-driver dependent, the drug response becomes an extreme scenario, in which F_1_ becomes 100% or nearly 100% and the second phase is eliminated. Analysis of cancer cell responses to dasatinib reveals that the ratio of biphasic versus monophasic responses is about 4:1. 

The biphasic response is consistent with multi-driver proliferation and the nature of targeted therapeutics. When a cancer cell has multiple independent proliferative drivers, if we had a perfect targeted drug that exclusively interacts with the driver alone, the drug would only partially inhibit cell proliferation, resulting in a shallow or partial inhibition. However, most targeted therapy agents are protein kinase inhibitors, and there are over 500 similarly structured protein kinases that are potential targets for a kinase inhibitor. Each kinase inhibitor displays a varying level of promiscuity toward the protein kinome. Most protein kinase inhibitors in clinical use have toxicity at some dose. Thus, biphasic inhibition is a natural manifestation of the unique characteristics of multi-driver proliferation, the precision focus of targeted therapy, and the promiscuity of targeted drugs. 

### 3.3. Biphasic Analysis Provides a Basis for Formulating Combination Targeted Therapy for Multi-Driver Cancers 

As noted above, the biphasic analysis breaks cancer cell–drug response into three quantitative parameters, F_1_/F_2_, K_d1_, and K_d2_, each representing a mechanistic aspect of the cell–drug interaction. Quantifying these mechanistic aspects, combined with knowledge of drug-target specificity and signaling pathways of targeted kinases, offers a rational basis for predicting effective drug combinations. When more than one drug with distinct F_1_ effects is identified against a cancer cell, the combination of these drugs has been found to be dramatically effective in blocking cell viability. Dose reduction of an order of magnitude or more is often achieved (Figure 5A). This has led to the identification of two-drug (BMS-754807 and AZD-6244) and three-drug (BMS-754807, AZD-6244 and dasatinib) combinations that are broadly effective against colorectal cancer cells. 

The CRC cell lines, HT-29, SK-CO-1, and NCI-H747 are potently inhibited by the combination of AZD-6244 and BMS-754807 with IC_50_s below 70 nM. The triple combination of AZD-6244/BMS-754807/dasatinib inhibited all three cell lines with IC_50_s at 40 nM or less (Figure 5B). These results, together with a previous report from this lab [31] indicate that most CRC cell lines are sensitive to the AZD-6244/BMS-754807 combination (7/11), and even more sensitive to the AZD-6244/BMS-754807/dasatinib combination (8/11). This level of potency is comparable to those of the most successful targeted therapy drugs for mono-driver cancers. Further in vivo and clinical investigations are needed to test the effectiveness of these drug combinations on CRC. 

It is yet to be tested how broadly applicable the biphasic analysis model is in predicting effective combination targeted therapy in vitro and in vivo beyond CRC. Multi-driver proliferation is believed to be broadly applicable to most cancers, because most cancers contain multiple driver mutations [4,5,6], and only a relatively small number of cancer subtypes can be treated effectively by mono-agent targeted therapy. However, this hypothesis needs to be directly and experimentally tested in broad cancer types. We have investigated two triple negative breast cancer cell lines and found that both relied on multiple drivers, and the biphasic mathematical model accurately describes their responses to targeted therapy, and predicts highly potent and strongly synergistic drug combinations [60]. Further investigations are needed to evaluate the clinical applicability of this approach.

Even though this current study focused on kinase-based target therapy agents, it is noted that targeted therapy can often be combined with chemotherapeutic agents to produce remarkable synergy [21]. It is possible that the synergistic targeted therapy combinations may be combined with chemotherapy to achieve the most favorable outcomes in clinical applications.

### 3.4. Biphasic Analysis Provides the Tool to Quantitatively Assess the Target-Specific and Off-Target Effects of Targeted Therapeutics 

Quantification of the toxicity effect using biphasic analysis is also potentially crucial in developing targeted therapy, especially for multi-driver cancers. Even though it is well recognized that protein kinase inhibitors tend to have off-target effects, which are likely the basis of clinical dose-limiting toxicity, it is difficult to distinguish the target-specific effects from the off-target effects. The IC_50_-based evaluation measures the combined sum of both effects. Consequently, off-target effect contributes to a lower IC_50_, which makes a drug appear more potent in vitro, but diminishes the effectiveness of a drug in the clinical applications. This Trojan horse characteristic of the off-target effect may have contributed to the discrepancy between in vitro studies and the clinical results of targeted therapy. The biphasic analysis offers a quantitative tool to evaluate the target-specific and off-target effects. The quantitative understanding of the two effects of a drug offers a better guide to laboratory and clinical applications of targeted therapy agents. 

## 4. Materials and Methods 

### 4.1. Cell Lines, Media and Drugs

All cell lines, HT-29, SK-CO-1, NCI-H747, and HCC-827, were purchased from ATCC (Manassas, VA, USA). ATCC authenticates all its cell lines by short tandem repeat profiling, cell morphology monitoring, karyotyping, and cytochrome C oxidase I testing. The cells were grown in ATCC-recommended media, containing 10% FBS and 1% Penicillin-streptomycin (Thermo Fisher Scientific, Pittsburgh, PA, USA). All cells were cultured at 37 °C in humid atmosphere containing 5% CO_2_. Kinase inhibitors were purchased from Selleckchem (Munich, Germany), LC Laboratories (Woburn, MA, USA) or AdooQ Bioscience (Irvine, CA, USA). 

### 4.2. Cell Culture and Viability Assays

Cell culture and viability assays were performed as described previously [18]. Briefly, cells were plated in 96-well plates at 25,000 cells per well, and were incubated with drugs at indicated concentrations in 100 µL medium containing 1% DMSO for 72 h. At the end of the incubation, 10 µL staining solution containing 5 mg/mL 3-(4,5-dimethylthiazol-2-yl)-2,5-diphenyltetrazolium bromide (MTT dye) (Thermo Fisher Scientific) was added into each well, and the plates were incubated at 37 °C for another 4 h. The medium in the wells was removed by aspiration, and DMSO (100 µL) was added into each well. After the purple formazon was dissolved, absorbance at 490 and 750 nm for each well was determined using a VersaMax Tunable Microplate Reader. The A490–A750 values were taken as indicators of cell viability. All cell growth and drug inhibition experiments were performed in triplicate at least three times. 

### 4.3. Drug Synergy Analysis and Combination Index Calculation

Drug synergy analysis was performed as described [31]. To determine if two drugs were synergistic in their inhibition of cell proliferation, cell viability was determined after incubating the cells in the presence of each drug alone or both drugs (1:1 ratio) at 16 concentrations ranging from 0.6 nM to 20 µM. The concentration of a drug or drug combination that inhibited cell viability at a certain percentage (IC_x_) was determined manually from the graphs of drug dose response curves. The dose reduction index (DRI) was calculated according to Chou [45], using the Chou—Talalay method [45]. Briefly, the IC_40_, IC_50_, IC_60_, etc., were determined from the dose response curves, and the DRI at a given level of inhibition was calculated by the following equation: DRI = IC_x−1_ × IC_x−2_/(IC_x−1+2_ × (IC_x−1_ + IC_x−2_), where IC_x−1_, IC_x−2_, and IC_x−1+2_ are the concentrations of drug 1, drug 2, and drugs 1 + 2, causing X% inhibition of cell viability, respectively. 

### 4.4. Curve Fitting by Single Target Equation and Biphasic Equation

Curve fitting was performed in Microsoft Excel. The equation for the single target binding is I = [D]/([D] + IC_50_), where I is the relative inhibition as the dependent variable, [D] is the drug concentration as the independent variable, IC_50_ is the drug concentration that inhibits viability by 50%, a constant to be determined through curve fitting. In curve fitting, the root mean square error (RMSE) between the experimentally determined cell viability and the calculated cell viability from the equation at each drug concentration was minimized using the IC_50_ as a variable. The IC_50_ that resulted in the best fit with the smallest RMSE was taken as the best fitting IC_50_. The RMSE minimization was carried out using the Solver add-in program in Microsoft Excel. 

The biphasic kinetic curve fitting was performed similarly, except that the equation used is a biphasic kinetic equation: I = F_1_ × [D]/([D] + K_d1_) + F_2_ × [D]/([D] + K_d2_). In this equation, the inhibition of cell viability (I) as a function of variable drug concentration is determined by four constants: F_1_ and F_2_, the phase 1 and phase 2 as fractions of total cell viability, K_d1_ and K_d2_, and the affinity of the drug for phase 1 and phase 2 targets. The equation assumed that the responses were biphasic, thus F_1_ and F_2_ added up to 1 (or 100%), so F_2_ = 1 − F_1_. The RMSE between the actual cell viability (1 − I) and the calculated cell viability from the biphasic equation as a function of the drug concentration was minimized, allowing the F_1_, K_d1_ and K_d2_ as variables. The F_1_, K_d1_ and K_d2_ that resulted in the smallest RMSE were taken as the best fitting parameters. F_2_ was calculated as 1 − F_1_.

The dose response data of HT-29 cells were also analyzed by a modified Hill equation: I = I_max_ × D^n^/(IC_50_*^n^ + D^n^). The meaning of the parameters and assumptions of this equation are discussed in the Results Section 2.2. Curve fitting to this equation, which minimized RMSE by varying I_max_, n and IC_50_*, was performed in Microsoft Excel using the Solver function. 

### 4.5. Western Blot Analysis of Drug Effects on Cell Signaling

To determine the effects of the kinase inhibitors on the signaling network in a given cell line, the cells were cultured to 70% confluency, and treated by the drugs at the indicated concentrations for 2 h. After the treatment, the culture medium was aspirated, and the cells were then resuspended in the SDS-PAGE loading buffer containing a protease inhibitor cocktail (Sigma-Aldrich, St Louis, MO, USA) and protein phosphatase inhibitors (PhosSTOP, Sigmal-Aldrich), and then lysed in a 95 °C heat bath immediately. SDS-PAGE and Western blotting were performed as described [31]. The loading was normalized based on the β-actin expression levels in the lysates determined by an antibody specific for this protein. All antibodies were purchased from Cell Signaling Technology (Danvers, MA, USA). The density of each protein band in the Western blots was measured using the ImageJ software (https://imagej.nih.gov/ij/).

### 4.6. Monophasic and Biphasic Analyses of Genomics of Drug Sensitivity in Cancer Cell Responses to Dasatinib

The dasatinib inhibition data (GDSC1-raw-data) were downloaded from the Genomics of Drug Sensitivity in Cancer website (https://www.cancerrxgene.org/downloads/bulk_download). The raw viability data were normalized to the controls as relative viability. In cases where the viability reading in the presence of low concentration drugs were higher than the controls, the highest reading was used as a control for normalizing the data. Monophasic and biphasic analyses were carried out as described in Section 4.4. 

## 5. Conclusions

Targeted therapy is an effective treatment for cancers that rely on a single proliferative driver. However, most cancers are sustained by multiple independent proliferative drivers. Such multi-driver cancer cells are only mildly inhibited by targeted therapy agents. This study revealed that the inhibition of several colorectal cancer cell lines by targeted therapy does not follow a traditional single target binding equation, and the potency cannot be represented by IC_50_. A biphasic mathematical model was developed that quantifies the drug response using three inhibitory parameters, ratio of target-specific inhibition, F_1_, potency of target-specific inhibition, K_d1_, and potency of off-target toxicity, K_d2_. This biphasic model and the mechanistic insights it reveals provide a new mechanistic perspective and mathematical tool for predicting effective combination targeted blockades against multi-driver cancer cells. Further in vivo and clinical investigations are required to validate this rational approach. 

The study identified an intriguing drug combination toward CRC. The CRC cell lines, HT-29, SK-CO-1, and NCI-H747, were potently inhibited by the combination of AZD-6244/ BMS-754807 (IC_50_s < 70 nM), and triple combination of AZD-6244/BMS-754807/dasatinib (IC_50_s < 40 nM). This level of potency is comparable to those of the most successful targeted therapy drugs for mono-driver cancers.

## Figures and Tables

**Table 1:** Inhibition of HT-29 viability by protein kinase inhibitors. Means ± SEM are reported for six sets of data from two independent experiments in triplicate. The table only lists the most common kinase targets for each inhibitor. Most of the inhibitors have been tested against most of the kinome, and complete inhibitory information is available at the Library of Integrated Network-based Cellular Signatures (http://lincs.hms.harvard.edu/kinomescan/).

Drug	Target	IC_50_ (µM)
HG6-64-1	BRAF	0.243 ± 0.02
Dasatinib	Src, Abl, PDGFR	0.403 ± 0.06
BMS-754807	IR, IGF-1R, Met	0.990 ± 0.09
AZD-6244	Mek	1.334 ± 0.18
Crizotinib	Met	8.326 ± 0.99
Sunitinib	PDGFRs	11.17 ± 0.61
BX-912	PDK1	12.82 ± 2.12
BGJ398	FGFRs	16.49 ± 1.71
Gefitinib	EGFR	>20
Pazopanib	PDGFR, VEGFR	>20
AZD-6482	PI 3-K	>20
Masitinib	PDGFR, DDR1, Abl	>20
Erlotinib	EGFR	>20
GSK-690693	Akt	>20
Imatinib	Abl	>20
Lapatinib	EGFR, ErbB2	>20
Linsitinib	IR, IGF-1R	>20
Neratinib	EGFRs	>20

**Table 2:** Inhibition of the HCC-827 cell line by protein kinase inhibitors. Means ± SEM are reported. The data were calculated using six sets of IC_50_ values from two experiments in triplicate.

Drug	Target	IC_50_ (nM)
Afatinib	EGFR, ErbB2/4	7.8 ± 0.2
Gefitinib	EGFR	12.1 ± 0.4
Erlotinib	EGFR	15.4 ± 0.5
Dasatinib	Src, Abl, PDGFRs, etc	140 ± 9
Bosutinib	Src, Abl,	273 ± 10
Neratinib	EGFR, ErbB2/3/4	512 ± 12
Lapatinib	ErbB2/3	585 ± 31
Sorafenib	DDR1/2, FLT3	3286 ± 44
Linifanib	PDGFRs	6871 ± 333
BMS-754807	IR, IGF-1R, Met	10,571 ± 797
Linsitinib	IR, IGF-1R	18,358 ± 1069
BGJ398	FGFRs	>20,000
Nilotinib	Abl, DDR1	>20,000
Sunitinib	PDGFRs	>20,000
Crizotinib	Met	>20,000
HG6-64-1	BRAF	>20,000
AZD-6244	Mek	>20,000
BX-912	PDK1	>20,000

## Supplementary Materials

The following are available online at https://www.mdpi.com/2072-6694/12/2/436/s1, Figure S1: Western blot analysis of total and phosphorylated insulin receptor and IGF-1R levels in HT-29 cells treated by different kinase inhibitors, Data S1: Biphasic analysis of cancer cell responses to dasatinib.

## Abbreviations

BP	biphasic
CML	chronic myeloid leukemia
CRC	colorectal cancer
DRI	dose reduction index
IGF-1R	insulin-like growth factor 1 receptor
IR	insulin receptor
MAP kinase	mitogen-activated protein kinase
MP	monophasic
PTK	protein tyrosine kinase
RMSE	root mean square error

## References

[1] Druker B.J. (2008). Translation of the Philadelphia chromosome into therapy for CML. Blood.

[2] Figueroa-Magalhães M.C., Jelovac D., Connolly R., Wolff A.C. (2014). Treatment of HER2-positive breast cancer. Breast.

[3] Da Cunha Santos G., Shepherd F.A., Tsao M.S. (2011). EGFR mutations and lung cancer. Annu. Rev. Pathol..

[4] Martincorena I., Raine K.M., Gerstung M., Dawson K.J., Haase K., Van Loo P., Davies H., Stratton M.R., Campbell P.J. (2017). Universal Patterns of Selection in Cancer and Somatic Tissues. Cell.

[5] Tomasetti C., Marchionni L., Nowak M.A., Parmigiani G., Vogelstein B. (2015). Only three driver gene mutations are required for the development of lung and colorectal cancers. Proc. Natl. Acad. Sci. USA.

[6] Vogelstein B., Papadopoulos N., Velculescu V.E., Zhou S., Diaz L.A., Kinzler K.W. (2013). Cancer genome landscapes. Science.

[7] Tolcher A.W., Peng W., Calvo E. (2018). Rational Approaches for Combination Therapy Strategies Targeting the MAP Kinase Pathway in Solid Tumors. Mol. Cancer Ther..

[8] Brenner H., Kloor M., Pox C.P. (2014). Colorectal cancer. Lancet.

[9] Loree J.M., Kopetz S. (2017). Recent developments in the treatment of metastatic colorectal cancer. Ther. Adv. Med. Oncol..

[10] Seeber A., Gastl G. (2016). Targeted Therapy of Colorectal Cancer. Oncol. Res. Treat..

[11] Bozic I., Antal T., Ohtsuki H., Carter H., Kim D., Chen S., Karchin R., Kinzler K.W., Vogelstein B., Nowak M.A. (2010). Accumulation of driver and passenger mutations during tumor progression. Proc. Natl. Acad. Sci. USA.

[12] Network C.G.A. (2012). Comprehensive molecular characterization of human colon and rectal cancer. Nature.

[13] Karoulia Z., Gavathiotis E., Poulikakos P.I. (2017). New perspectives for targeting RAF kinase in human cancer. Nat. Rev. Cancer.

[14] Frame M.C. (2002). Src in cancer: Deregulation and consequences for cell behaviour. Biochim. Biophys. Acta.

[15] Johnson F.M., Gallick G.E. (2007). SRC family nonreceptor tyrosine kinases as molecular targets for cancer therapy. Anticancer Agents Med. Chem..

[16] Zhang S., Yu D. (2012). Targeting Src family kinases in anti-cancer therapies: Turning promise into triumph. Trends Pharmacol. Sci..

[17] Puls L.N., Eadens M., Messersmith W. (2011). Current status of SRC inhibitors in solid tumor malignancies. Oncologist.

[18] O’Neil J., Benita Y., Feldman I., Chenard M., Roberts B., Liu Y., Li J., Kral A., Lejnine S., Loboda A. (2016). An Unbiased Oncology Compound Screen to Identify Novel Combination Strategies. Mol. Cancer Ther..

[19] Wali V.B., Langdon C.G., Held M.A., Platt J.T., Patwardhan G.A., Safonov A., Aktas B., Pusztai L., Stern D.F., Hatzis C. (2017). Systematic Drug Screening Identifies Tractable Targeted Combination Therapies in Triple-Negative Breast Cancer. Cancer Res..

[20] Menden M.P., Wang D., Mason M.J., Szalai B., Bulusu K.C., Guan Y., Yu T., Kang J., Jeon M., Wolfinger R. (2019). Community assessment to advance computational prediction of cancer drug combinations in a pharmacogenomic screen. Nat. Commun..

[21] Holbeck S.L., Camalier R., Crowell J.A., Govindharajulu J.P., Hollingshead M., Anderson L.W., Polley E., Rubinstein L., Srivastava A., Wilsker D. (2017). The National Cancer Institute ALMANAC: A Comprehensive Screening Resource for the Detection of Anticancer Drug Pairs with Enhanced Therapeutic Activity. Cancer Res..

[22] Fitzgerald J.B., Schoeberl B., Nielsen U.B., Sorger P.K. (2006). Systems biology and combination therapy in the quest for clinical efficacy. Nat. Chem. Biol..

[23] Palmer A.C., Sorger P.K. (2017). Combination Cancer Therapy Can Confer Benefit via Patient-to-Patient Variability without Drug Additivity or Synergy. Cell.

[24] Fallahi-Sichani M., Honarnejad S., Heiser L.M., Gray J.W., Sorger P.K. (2013). Metrics other than potency reveal systematic variation in responses to cancer drugs. Nat. Chem. Biol..

[25] Shoemaker R.H. (2006). The NCI60 human tumour cell line anticancer drug screen. Nat. Rev. Cancer.

[26] Forbes S.A., Beare D., Boutselakis H., Bamford S., Bindal N., Tate J., Cole C.G., Ward S., Dawson E., Ponting L. (2017). COSMIC: Somatic cancer genetics at high-resolution. Nucleic Acids Res..

[27] Windham T.C., Parikh N.U., Siwak D.R., Summy J.M., McConkey D.J., Kraker A.J., Gallick G.E. (2002). Src activation regulates anoikis in human colon tumor cell lines. Oncogene.

[28] Herr R., Köhler M., Andrlová H., Weinberg F., Möller Y., Halbach S., Lutz L., Mastroianni J., Klose M., Bittermann N. (2015). B-Raf inhibitors induce epithelial differentiation in BRAF-mutant colorectal cancer cells. Cancer Res..

[29] Liu G., Song Y., Cui L., Wen Z., Lu X. (2015). Inositol hexaphosphate suppresses growth and induces apoptosis in HT-29 colorectal cancer cells in culture: PI3K/Akt pathway as a potential target. Int. J. Clin. Exp. Pathol..

[30] Lee H.S., Cho H.J., Kwon G.T., Park J.H. (2014). Kaempferol Downregulates Insulin-like Growth Factor-I Receptor and ErbB3 Signaling in HT-29 Human Colon Cancer Cells. J. Cancer Prev..

[31] Shen J., Li L., Yang T., Cheng N., Sun G. (2019). Drug Sensitivity Screening and Targeted Pathway Analysis Reveal a Multi-Driver Proliferative Mechanism and Suggest a Strategy of Combination Targeted Therapy for Colorectal Cancer Cells. Molecules.

[32] Li L., Cui Y., Shen J., Dobson H., Sun G. (2019). Evidence for activated Lck protein tyrosine kinase as the driver of proliferation in acute myeloid leukemia cell, CTV-1. Leuk. Res..

[33] Gray N.S. (2011). Type II Raf Kinase Inhibitors. International Patent Number.

[34] Bernabé R., Patrao A., Carter L., Blackhall F., Dean E. (2016). Selumetinib in the treatment of non-small-cell lung cancer. Future Oncol..

[35] Araujo J., Logothetis C. (2010). Dasatinib: A potent SRC inhibitor in clinical development for the treatment of solid tumors. Cancer Treat. Rev..

[36] Carboni J.M., Wittman M., Yang Z., Lee F., Greer A., Hurlburt W., Hillerman S., Cao C., Cantor G.H., Dell-John J. (2009). BMS-754807, a small molecule inhibitor of insulin-like growth factor-1R/IR. Mol. Cancer Ther..

[37] Rhodes N., Heerding D.A., Duckett D.R., Eberwein D.J., Knick V.B., Lansing T.J., McConnell R.T., Gilmer T.M., Zhang S.Y., Robell K. (2008). Characterization of an Akt kinase inhibitor with potent pharmacodynamic and antitumor activity. Cancer Res..

[38] Manning B.D., Toker A. (2017). AKT/PKB Signaling: Navigating the Network. Cell.

[39] Lee S., Lin X., Nam N.H., Parang K., Sun G. (2003). Determination of the substrate-docking site of protein tyrosine kinase C-terminal Src kinase. Proc. Natl. Acad. Sci. USA.

[40] Tiwari R.K., Brown A., Sadeghiani N., Shirazi A.N., Bolton J., Tse A., Verkhivker G., Parang K., Sun G. (2017). Design, Synthesis, and Evaluation of Dasatinib-Amino Acid and Dasatinib-Fatty Acid Conjugates as Protein Tyrosine Kinase Inhibitors. ChemMedChem.

[41] Levy D.S., Kahana J.A., Kumar R. (2009). AKT inhibitor, GSK690693, induces growth inhibition and apoptosis in acute lymphoblastic leukemia cell lines. Blood.

[42] Altomare D.A., Zhang L., Deng J., Di Cristofano A., Klein-Szanto A.J., Kumar R., Testa J.R. (2010). GSK690693 delays tumor onset and progression in genetically defined mouse models expressing activated Akt. Clin Cancer Res..

[43] Chan T.O., Tsichlis P.N. (2001). PDK2: A complex tail in one Akt. Sci. Signal..

[44] Sarbassov D.D., Guertin D.A., Ali S.M., Sabatini D.M. (2005). Phosphorylation and regulation of Akt/PKB by the rictor-mTOR complex. Science.

[45] Chou T.C. (2010). Drug combination studies and their synergy quantification using the Chou-Talalay method. Cancer Res..

[46] Guagnano V., Furet P., Spanka C., Bordas V., Le Douget M., Stamm C., Brueggen J., Jensen M.R., Schnell C., Schmid H. (2011). Discovery of 3-(2,6-dichloro-3,5-dimethoxy-phenyl)-1-{6-[4-(4-ethyl-piperazin-1-yl)-phenylamino]-pyrimidin-4-yl}-1-methyl-urea (NVP-BGJ398), a potent and selective inhibitor of the fibroblast growth factor receptor family of receptor tyrosine kinase. J. Med. Chem..

[47] Nishii H., Chiba T., Morikami K., Fukami T.A., Sakamoto H., Ko K., Koyano H. (2010). Discovery of 6-benzyloxyquinolines as c-Met selective kinase inhibitors. Bioorg. Med. Chem. Lett..

[48] Tsao M.S., Sakurada A., Cutz J.C., Zhu C.Q., Kamel-Reid S., Squire J., Lorimer I., Zhang T., Liu N., Daneshmand M. (2005). Erlotinib in lung cancer—Molecular and clinical predictors of outcome. N. Engl. J. Med..

[49] Amann J., Kalyankrishna S., Massion P.P., Ohm J.E., Girard L., Shigematsu H., Peyton M., Juroske D., Huang Y., Stuart Salmon J. (2005). Aberrant epidermal growth factor receptor signaling and enhanced sensitivity to EGFR inhibitors in lung cancer. Cancer Res..

[50] Baro M., de Llobet L.I., Figueras A., Skvortsova I., Mesia R., Balart J. (2014). Dasatinib worsens the effect of cetuximab in combination with fractionated radiotherapy in FaDu- and A431-derived xenografted tumours. Br. J. Cancer.

[51] Zhang J., Kalyankrishna S., Wislez M., Thilaganathan N., Saigal B., Wei W., Ma L., Wistuba I.I., Johnson F.M., Kurie J.M. (2007). SRC-family kinases are activated in non-small cell lung cancer and promote the survival of epidermal growth factor receptor-dependent cell lines. Am. J. Pathol..

[52] Neuber S., Jäger S., Meyer M., Wischmann V., Koch P.J., Moll R., Schmidt A. (2015). c-Src mediated tyrosine phosphorylation of plakophilin 3 as a new mechanism to control desmosome composition in cells exposed to oxidative stress. Cell Tissue Res..

[53] Di Veroli G.Y., Fornari C., Goldlust I., Mills G., Koh S.B., Bramhall J.L., Richards F.M., Jodrell D.I. (2015). An automated fitting procedure and software for dose-response curves with multiphasic features. Sci. Rep..

[54] Iorio F., Knijnenburg T.A., Vis D.J., Bignell G.R., Menden M.P., Schubert M., Aben N., Gonçalves E., Barthorpe S., Lightfoot H. (2016). A Landscape of Pharmacogenomic Interactions in Cancer. Cell.

[55] Ellis L.M., Hicklin D.J. (2009). Resistance to Targeted Therapies: Refining Anticancer Therapy in the Era of Molecular Oncology. Clin. Cancer Res..

[56] Corso S., Giordano S. (2014). Targeted therapies in cancer and mechanisms of resistance. J. Mol. Med..

[57] Neel D.S., Bivona T.G. (2017). Resistance is futile: Overcoming resistance to targeted therapies in lung adenocarcinoma. NPJ Precis. Oncol..

[58] Gorre M.E., Mohammed M., Ellwood K., Hsu N., Paquette R., Rao P.N., Sawyers C.L. (2001). Clinical resistance to STI-571 cancer therapy caused by BCR-ABL gene mutation or amplification. Science.

[59] Prahallad A., Sun C., Huang S., Di Nicolantonio F., Salazar R., Zecchin D., Beijersbergen R.L., Bardelli A., Bernards R. (2012). Unresponsiveness of colon cancer to BRAF(V600E) inhibition through feedback activation of EGFR. Nature.

[60] Sun G. (2020). Biphasic Analysis of Drug Response to Targeted Therapy by Triple Negative Breast Cancer Cells.

