# Supplementary material for: Biphasic Mathematical Model of Cell–Drug Interaction That Separates Target-Specific and Off-Target Inhibition and Suggests Potent Targeted Drug Combinations for Multi-Driver Colorectal Cancer Cells
Source: Cancers (Basel). 2020 Feb 13;12(2):436. doi: 10.3390/cancers12020436 (PMC7072552; doi:10.3390/cancers12020436)
Supplement: Supplementary file 1 [file cancers-12-00436-s001.zip › Revised Shen et al. Supplementary Materials Figs and Tables.pdf]

Supplementary Materials 1:

Fig. S1. Analysis of HT-29 dose-dependent inhibition by protein kinase inhibitors. The dose response data (closed circles) of HT-29 cells to AZD-6244 (A), BMS-754807 (B), dasatinib (C), and HG6-64-1 (D) were curve-fitted to the monophasic kinetic equation $I = [D] / ([D] + IC_{50})$ (dashed line) and the Hill equation $I = I_{max} \times D^n/(IC_{50}{*}^n+D^n)$ (solid line). The parameters obtained from curve-fitting are presented in the graphs (dashed line parameters in the upper right corners, and the solid line parameters in the bottom left corners).

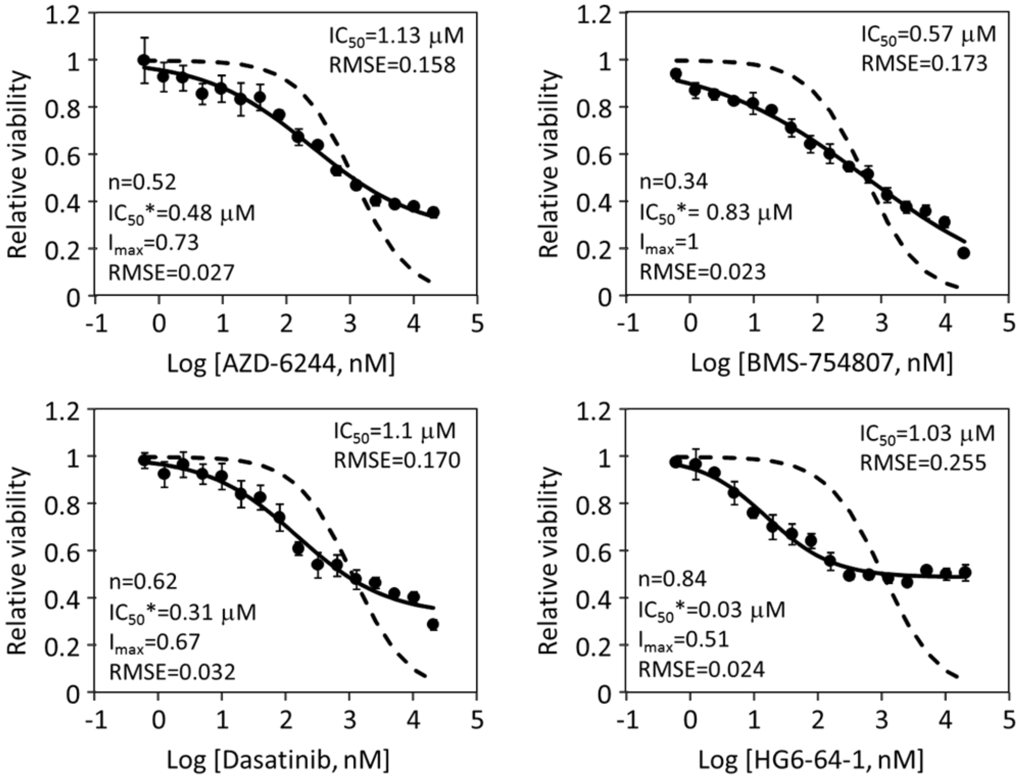

Fig. S2. Full pictures of Western blot analyses of selected kinase inhibitors on HT-29 cell line signaling. These are the full pictures of the Western blot results used to generate Fig. 2C.

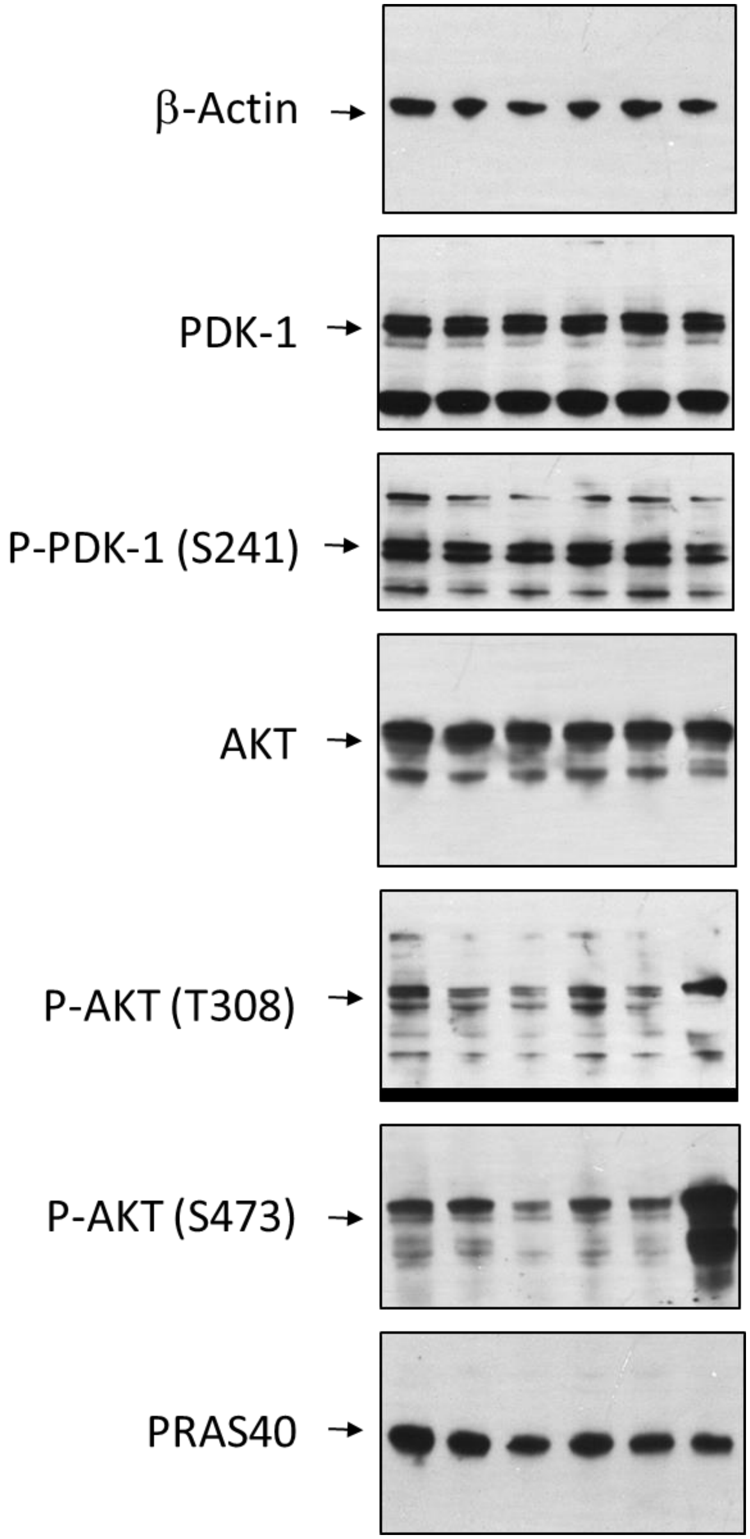

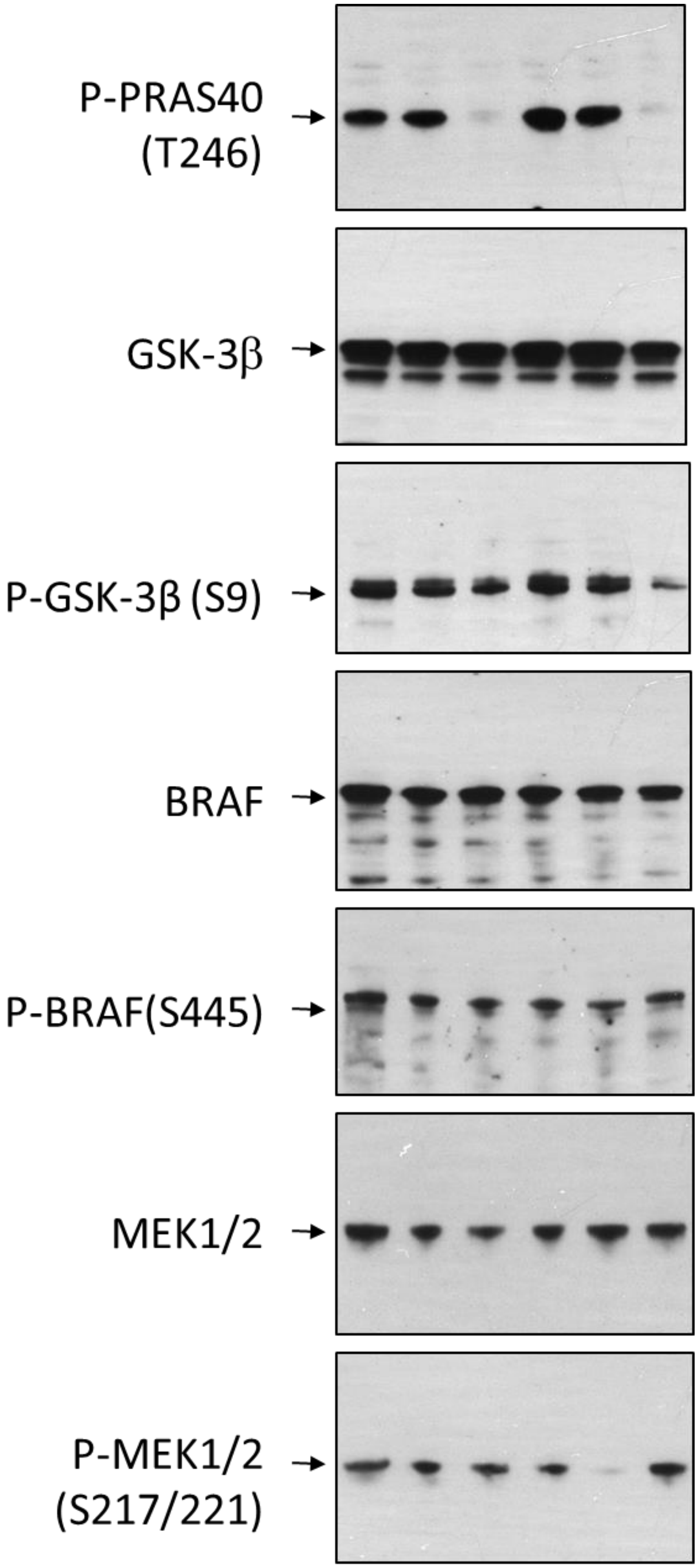

P-PRAS40 (T246)
GSK-3β
P-GSK-3β (S9)
BRAF
P-BRAF(S445)
MEK1/2
P-MEK1/2 (S217/221)

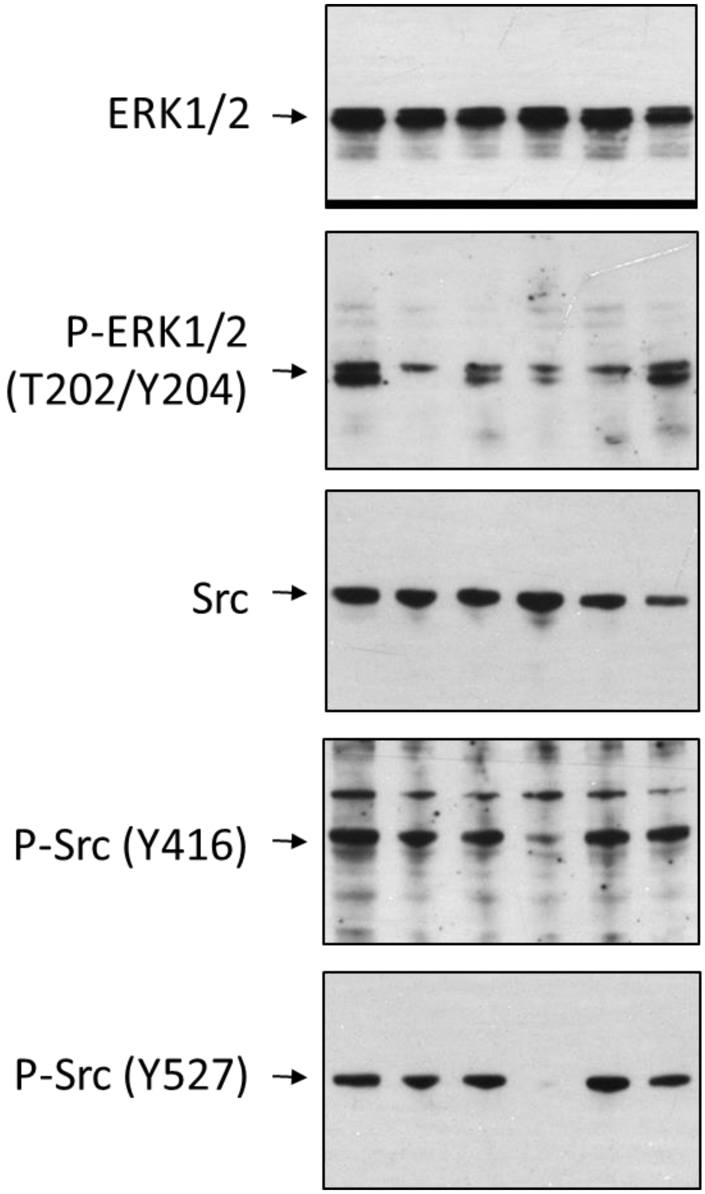
ERK1/2
P-ERK1/2
(T202/Y204)
Src
P-Src (Y416)
P-Src (Y527)

Fig. S3. Western blot analysis of total and phosphorylated insulin receptor and IGF-1R levels in HT-29 cells treated by different kinase inhibitors. Treatment and Western blot analysis were carried out as described in the legend to Fig 2. In each image, the top band corresponds to the size of the receptor.

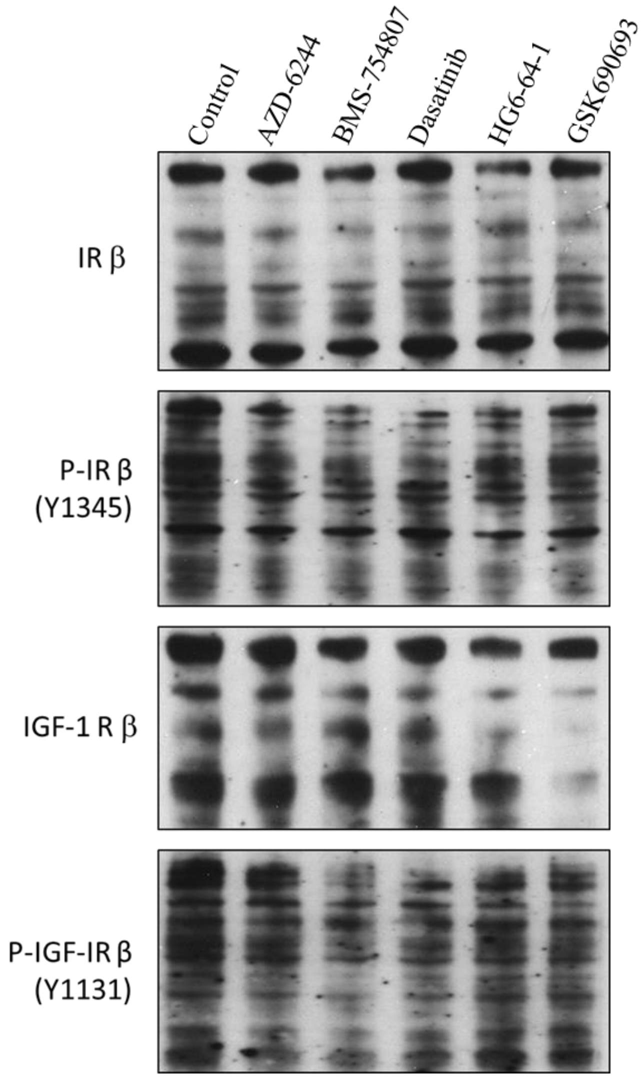

Fig. S4. Full pictures of Western blot analyses of selected kinase inhibitors on HCC-827 cell line signaling. These are the full pictures of the Western blot results used to generate Fig. 6F.

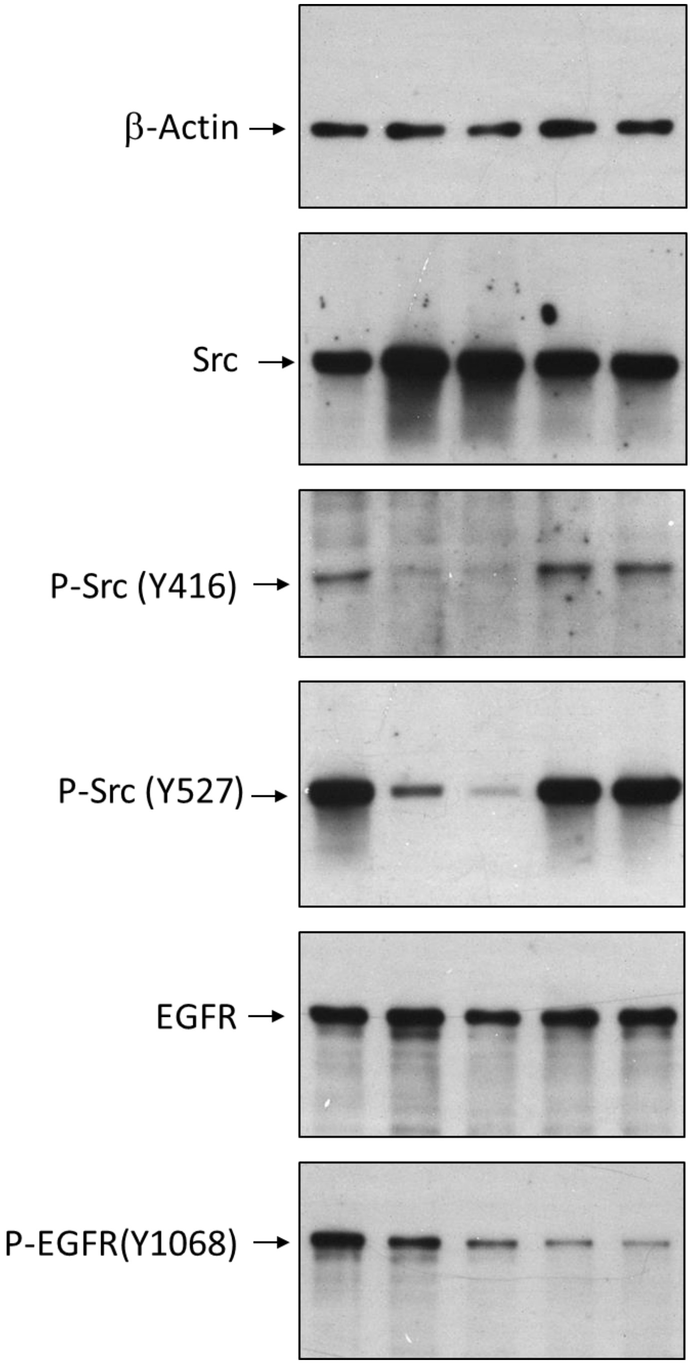

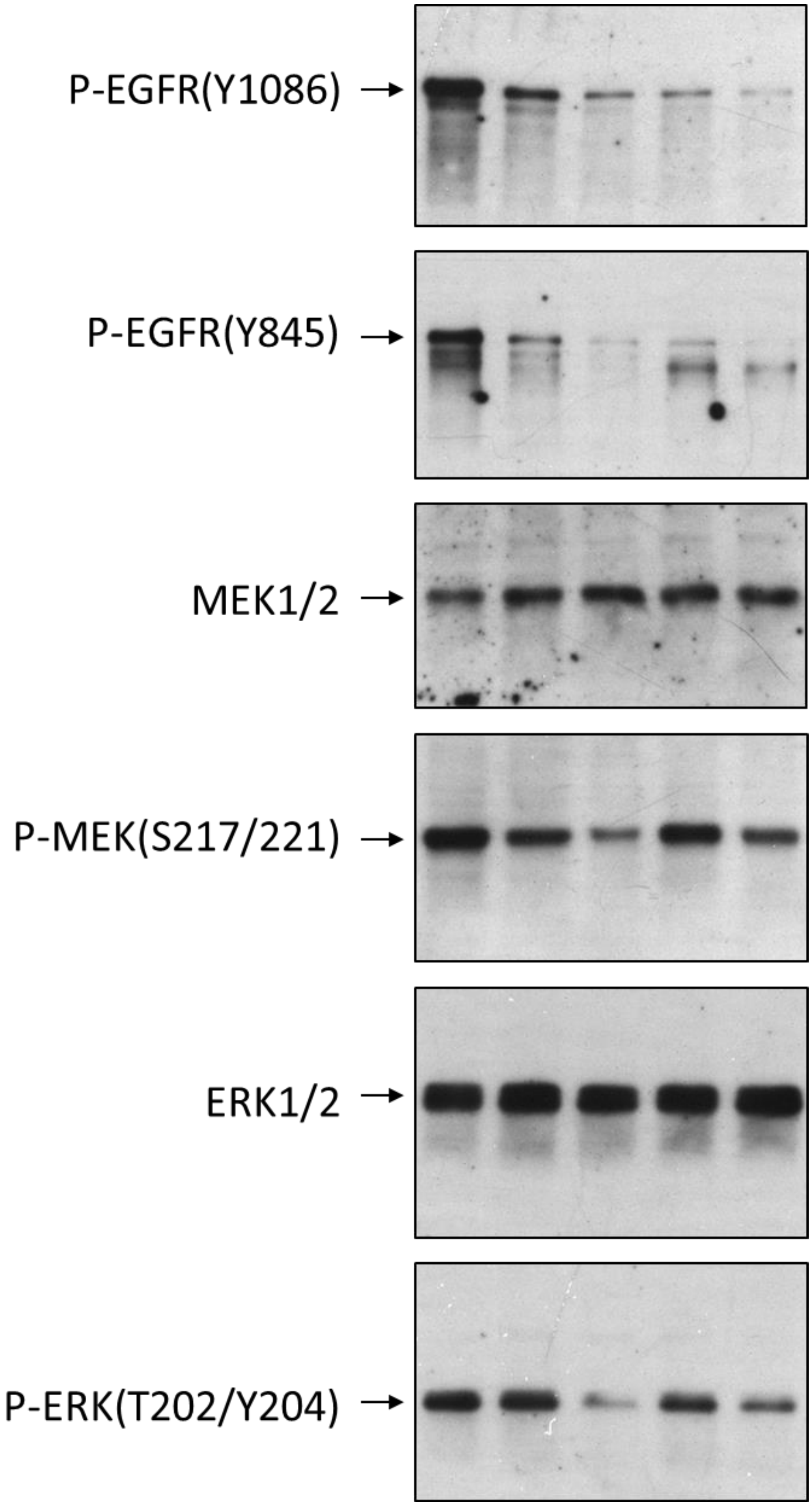
P-EGFR(Y1086)
P-EGFR(Y845)
MEK1/2
P-MEK(S217/221)
ERK1/2
P-ERK(T202/Y204)

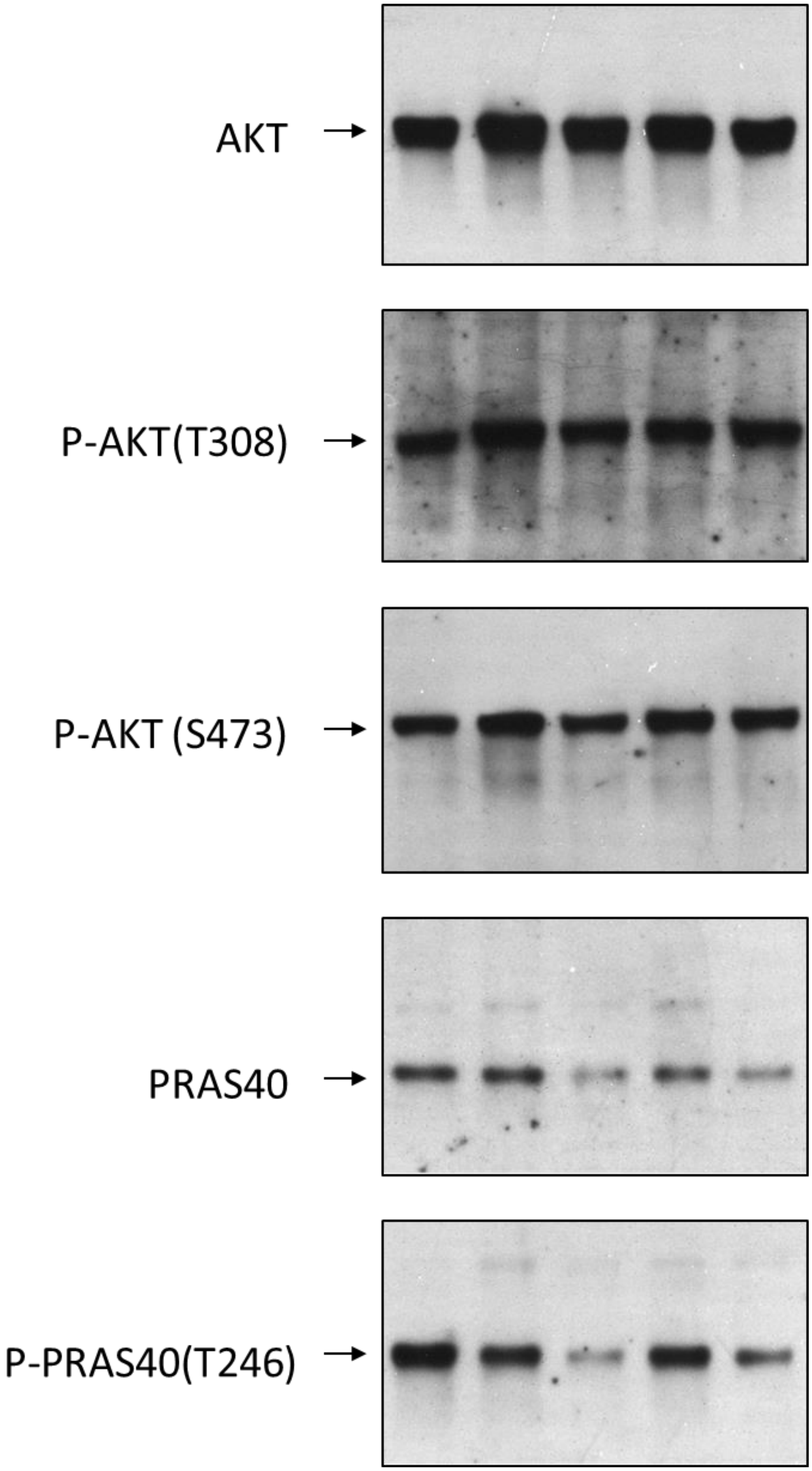
AKT
P-AKT(T308)
P-AKT (S473)
PRAS40
P-PRAS40(T246)

Table S1. Densitometry analyses of the band density presented in Fig. 2C. The densities of appropriate bands in 2C were analyzed using the ImageJ software. For each protein, the six bands for all treatments were analyzed using the same analysis box containing the whole band. The densities were compared to the control (no drug treatment) and normalized to the loading control, β-actin. The number above 2 is labeled green, and the numbers below 0.5 are labeled red.

	Control	AZD-6244	BMS-754807	Dasatinib	HG6-64-1	GSK690693
β-actin	1	1	1	1	1	1
PDK-1	1	1.046104	1.159111	1.147543	1.059144	1.034902
P-PDK-1 (S241)	1	1.072759	1.122446	1.156274	1.005551	0.904825
AKT	1	1.131456	1.20131	1.20525	0.980167	1.177483
pAKT(T308)	1	0.652152	0.484466	0.998284	0.452613	1.406799
pAKT(S473)	1	1.20339	0.707887	1.124539	0.823294	2.463198
PRAS40	1	1.095238	0.947064	1.078976	0.859161	0.876639
P-PRAS40 (T246)	1	1.308334	0.253356	1.644941	1.27588	0.163201
GSK-3β	1	1.207881	1.250831	1.28183	1.085261	1.100336
P-GSK-3β (S9)	1	1.0629	0.838327	1.254432	0.962482	0.451151
BRAF	1	0.995861	1.112981	1.022657	0.825885	0.912292
P-BRAF (S445)	1	0.794982	0.860506	0.743535	0.564455	0.928068
MEK 1/2	1	0.872611	0.858911	0.985739	0.944587	1.176941
P-MEK 1/2 (S217/221)	1	0.971807	1.062488	0.860634	0.209916	1.261475
ERK 1/2	1	1.139803	1.174971	1.233042	1.059567	1.040788
P-ERK (T202/Y204)	1	0.386077	0.730268	0.484445	0.510203	1.136269
Src	1	1.130438	1.191662	1.426501	0.898991	0.745081
P-Src (Y416)	1	0.858366	0.993353	0.469683	0.924531	0.942216
P-Src (Y527)	1	1.01375	1.334623	0.223951	1.214101	1.054115

Table S2. Densitometry analyses of the band density presented in Fig. 6F. The densities of appropriate bands in Fig. 6F were analyzed using the ImageJ software. For each protein, the five bands for all treatments were analyzed using the same analysis box containing the whole band. The densities were compared to the control (no drug treatment) and normalized to the loading control, β-actin. The numbers below 0.5 are labeled red.

	Control	Dasa400	Dasa800	Gef50	Gef100
β-actin	1	1	1	1	1
Src	1	1.454739	1.812509	1.143042	1.291881
p-Src (Y416)	1	0.7168	0.944469	1.163292	1.193636
P-Src (Y527)	1	0.239669	0.070064	0.805882	0.99858
EGFR	1	1.16889	0.978319	0.86922	1.072395
P-EGFR (Y1068)	1	0.678677	0.408403	0.17699	0.208516
P-EGFR (Y1086)	1	0.458206	0.212859	0.07919	-0.05193
P-EGFR (Y845)	1	0.400548	0.089934	0.115503	-0.01864
MEK 1/2	1	1.194331	1.567552	1.04764	1.218064
P-MEK 1/2 (S217/S221)	1	0.719851	0.452104	0.692377	0.473383
ERK 1/2	1	1.137153	1.341201	0.962051	1.240311
P-ERK 1/2 (T202/Y204)	1	0.973838	0.341771	0.684984	0.465598
AKT	1	1.117882	1.312981	0.944923	1.001151
P-AKT (T308)	1	1.158578	1.29545	0.903416	1.086385
PAKT (S473)	1	1.170423	1.219307	1.034177	1.104584
PRAS40	1	1.007558	0.412679	0.628671	0.288923
P-PRAS40 (T246)	1	0.693183	0.210193	0.624464	0.259739
